# Determining the molecular and physiological actions of subtype-selective nanobodies of GABA_A_ receptors

**DOI:** 10.1126/sciadv.aeg3548

**Published:** 2026-07-29

**Authors:** Jose Enrique Gonzalez-Prada, Sulin Liu, Chuhan Shang, Chloe S. Chernoff, Damian P. Bright, Martin Mortensen, Charlotte F. Jones, Stephanie Nestorow, Vikram Babu Kasaragod, Wan-Na Chen, Saad Hannan, Jianchong Zhou, Alexander W. E. Dunn, Asma Soltani, Richard J. Turner, Natasha M. Duggan, Yin Yuan, Ayla A. Wahid, Steven W. Hardwick, Suzanne Scott, Dimitri Y. Chirgadze, Els Pardon, Jan Steyaert, A. Radu Aricescu, Ole Paulsen, David Belin, Trevor G. Smart, Paul S. Miller

**Affiliations:** ^1^ Department of Pharmacology, University of Cambridge, Cambridge, UK.; ^2^ Department of Psychology, University of Cambridge, Cambridge, UK.; ^3^ Department of Neuroscience, Physiology and Pharmacology, University College London, London, UK.; ^4^ MRC Laboratory of Molecular Biology, Cambridge Biomedical Campus, Cambridge, UK.; ^5^ Department of Molecular and Cellular Biology, Harvard University, Cambridge, MA, USA.; ^6^ Department of Physiology, Development and Neuroscience, University of Cambridge, Cambridge, UK.; ^7^ CryoEM Facility, Department of Biochemistry, University of Cambridge, Cambridge, UK.; ^8^ Structural Biology Brussels, Vrije Universiteit Brussel (VUB), Brussels, Belgium.; ^9^ VIB-VUB Center for Structural Biology, VIB, Brussels, Belgium.

## Abstract

γ-Aminobutyric acid type-A (GABA_A_) receptors are the principal mediators of inhibitory neurotransmission in the human central nervous system. The α_2_- and α_3_-containing subtypes have tightly controlled spatial expression profiles, which influence anxiety, nociception, epilepsy, and autism. α_2_/α_3_-Selective small molecules compromise on strength of effect (efficacy) to avoid off-subtype modulation. To break this pharmacological deadlock, we study here a panel of nanobodies (NBs) raised against α_2_- and α_3_-containing GABA_A_ receptors. We identify subtype selective silent binders, positive allosteric modulators (PAMs), and inhibitors. Cryo–electron microscopy structures explain the binding modes and molecular mechanisms of action of representative NBs. Modulators exhibit distinct synaptic and extrasynaptic functional profiles in brain slices and neuronal networks and can reduce anxiety in vivo. These selective and efficacious NBs (whether inhibitors or positive modulators) enable strong yet precise pharmacological control of α_2_/α_3_-containing subtypes to advance basic research and as potential therapeutic leads to treat neuropsychiatric disorders.

## Teaser

Nanobodies modulate GABA_A_ α_2_/α_3_ receptors selectively and efficaciously to boost neuronal inhibition and exert anxiolysis.

## INTRODUCTION

γ-Aminobutyric acid type-A (GABA_A_) receptors are pentameric ligand-gated ion channels that assemble from 19 possible subunits (α_1–6_, β_1–3_, γ_1–3_, δ, ε, θ, π, and ρ_1–3_), with the most abundant isoform comprising two α, two β, and one γ subunit (*1*, *2*). Each subunit comprises a β-sandwich extracellular domain (ECD), a four α-helical transmembrane domain (TMD), and a structurally undefined ∼100– to 150–amino acid M3-M4 intracellular domain (ICD). GABA binds to an orthosteric site at both β-α ECD interfaces to trigger downstream channel activation and influx of Cl^−^ ions that promote neuronal membrane hyperpolarization (*3*, *4*).

GABA_A_ receptor dysfunction is associated with various neurological and psychiatric conditions, including autism, epilepsy, anxiety, schizophrenia, depression, and substance use disorder (*5*). Benzodiazepines (BZDs) constitute a widely prescribed class of drugs that exert hypnotic-anxiolytic effects by acting as positive allosteric modulators (PAMs) of GABA_A_ receptors. However, BZDs bind the extracellular α-γ interface indiscriminately across the major α_1/2/3/5_-γ_2_–containing receptor subtypes, contributing to side effects, such as sedation, amnesia, muscle weakness, tolerance, and addiction, rendering them unsuitable for long-term use (*6*). Alternative modulators that instead bind the TMD, such as intravenous anesthetics and neurosteroids, are also nonselective between the α-containing subtypes (*2*). α-subunit genetically modified mice and/or ligands have demonstrated beneficial roles for α_2_/α_3_-containing subtypes in anxiety (*7*–*10*), nociception (*11*, *12*), epilepsy (*13*, *14*), and autism (*15*) while mitigating sedating, amnesiac, and addictive effects. However, the high degree of amino acid identity within the α/γ pocket (*16*) means small-molecule screens have been unsuccessful in identifying ligands selective by binding against α_2_/α_3_-containing subtypes. Instead, selectivity has relied upon differential modulatory efficacy (*7*–*9*, *11*, *13*–*15*), which coincides with reducing the maximum PAM effect until it no longer manifests through undesirable subtypes. Thus, an affinity versus efficacy deadlock has complicated α_2_/α_3_ small-molecule design, limiting therapeutic leads (*17*).

Nanobodies (NBs) are Variable Heavy domain of Heavy-chain antibody (VHH) domains from camelid antibodies, and variants have been developed that can modulate ion channels (*18*–*20*). They form larger contact zones to targets than small molecules, ∼1000 Å^2^ versus ∼300 Å^2^ (*21*, *22*), making them more likely to form interactions with subtype-specific amino acids. Recently, antibodies, NBs, and toxins against α_1_β_2/3_-containing GABA_A_ receptors capable of PAM effects or inhibition have been identified (*23*–*25*). As pharmacological tools to study the role of specific GABA_A_ receptor subtypes in physiology and neuropsychiatric disorders, NBs offer easy in-house production, engineering, and conjugation with tracking dyes (*26*–*28*). Furthermore, NBs can modulate central nervous system (CNS) neurotransmitter receptors to treat behavioral phenotypes in mouse disease models (*29*). Therapeutically, NBs, such as caplacizumab (*30*), are clinically proven, and systemic brain shuttles can deliver NB cargo to the CNS (*31*).

Here, we report the actions of NBs raised in llamas against human GABA_A_ receptor antigens containing α_2_β_3_ or α_3_β_3_ subunits. We show examples of subtype selective silent binders, PAMs, and inhibitors and explain mechanisms of action through binding assays, cryo–electron microscopy (cryo-EM) structure determination and electrophysiology. Selected NBs can modulate both synaptic and extrasynaptic GABAergic neuronal signaling in acute brain slices and network activity in neuronal cultures and exert anxiolytic effects in rats as assessed in the elevated plus maze (EPM) (*32*).

## RESULTS

### α_2_ and α_3_ NBs bind to cell surface GABA_A_ receptors

**Fig. 1.**
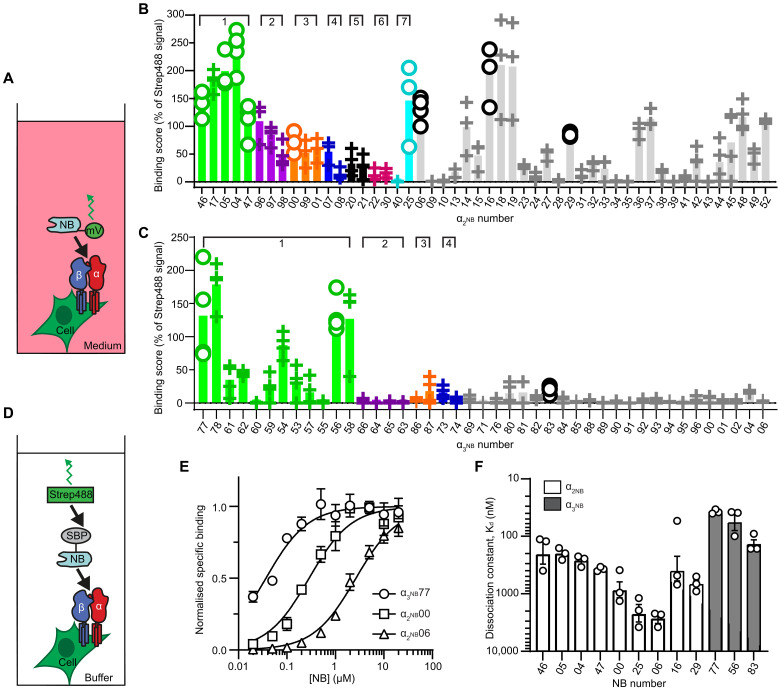
α_2_ and α_3_ NBs bind to GABA_A_ receptors expressed at the cell surface. (**A**) Schematic of single well from 96-well plate for screening NB-mV medium against cells expressing α_2_β_3_ or α_3_β_3_ receptors. (**B** and **C**) Binding scores (mean) for NB-mVs against (B) α_2_β_3_ and (C) α_3_β_3_ receptors versus streptavidin-AF488 labeling of SBP-β_3_ subunits (*n* = 3). Brackets above bars indicate NB family number. Circle symbols denote NBs characterized in more detail in this study. (**D**) Binding assay schematic using purified SBP-NB at known concentration, labeled by secondary binder streptavidin-AF488. (**E**) Example NB specific binding curves in the presence of 500 μM GABA. Mean ± SEM (*n* = 3). (**F**) Summary histogram showing NB binding dissociation constants (*K*_d_ values) in the presence of 500 μM GABA. Mean ± SEM (*n* = 3).

α_2_β_3_ or α_3_β_3_ receptors lacking the ICD to target NBs against the ECD were used in llama immunizations, and 50 α_2NB_ binders (00-52 and 96-99) and 43 α_3NB_ binders (53-99 and 00-06) with unique sequences were identified (see Materials and Methods). The utility of these NBs as physiological tools and therapeutics is dependent on effective binding to GABA_A_ receptors in cell membranes. To test this, we expressed NBs fused with C-terminal monoVenus (mV) (*33*) from Expi293F cells. Concentrations in the conditioned media were 1 to 12 μM when titrated by fluorescence against a purified NB-mV in blank conditioned medium, and NB-mV was the predominant SDS–polyacrylamide gel electrophoresis protein band (fig. S1A). The expression media were screened on human embryonic kidney (HEK) 293T cells expressing either α_2_β_3_ or α_3_β_3_ GABA_A_ receptors. The binding signal was normalized versus streptavidin–Alexa Fluor 488 (AF488) labeling of streptavidin-binding protein (SBP) fused to the GABA_A_ receptor β_3_ subunit (Fig. 1, A to C). Applying a 10% binding signal cutoff, many more α_2_ NBs bound α_2_ receptors than α_3_ NBs bound α_3_ receptors: ^42^/_50_ (84%) versus ^16^/_43_ (37%), respectively. On the basis of sequence similarity of complementarity-determining region 3 (CDR3), α_2NB_ and α_3NB_ binders clustered into 38 and 27 families, respectively (Fig. 1, A and B, and fig. S1, B and C). For the three largest α_2NB_ CDR3 families 1–3, binding scores were consistent within each family, although there were differences within two of the two-member families: α_2NB_07 versus α_2NB_08 (family 4); α_2NB_25 versus α_2NB_40 (family 7) (Fig. 1B). In contrast, the largest α_3NB_ CDR3 family 1 exhibited highly variable intrafamily binding scores despite similar CDR3 sequences (Fig. 1C). Despite α_2_- and α_3_-containing subtypes retaining the same architecture and highly similar structure [root mean square deviation (RMSD) Cα α_2_ from α_2_β_3_-α_2NB_06 versus α_3_ from α_3_β_3_-α_3NB_77 ECDs = 1.2 Å; structures described later], the NB repertoire against the two subtypes differed markedly in CDR3 length (fig. S1D): α_2NB_ range = 6 to 21 residues, mean = 15 residues; α_3NB_ range = 2 to 19 residues, and mean = 9 residues. CDR3 length versus binding score revealed a moderate positive correlation for α_2NB_ binders but no correlation for α_3NB_ binders (Spearman’s rank correlation, respectively: *r*_s_ = 0.49, *n* = 50, *P* < 0.001; *r*_s_ = 0.04, *n* = 43, *P* = 0.78) (fig. S1, E and F).

To evaluate selectivity, the 42 confirmed α_2NB_ binders to α_2_ and the 16 confirmed α_3NB_ binders to α_3_ were screened against the alternative α-containing subtypes. Only two α_2NB_ binders cross-reacted by >5%, both against α_1_: α_2NB_29 (∼7%) and α_2NB_41 (∼15%) (table S1). Two α_3NB_ binders also cross-reacted: α_3NB_56 against the α_6_-containing subtype (∼25%) and α_3NB_73 against the α_5_-containing subtype (90%), with the latter being stronger than its binding to the original α_3_-containing subtype (∼16%) (table S1). To evaluate affinity, we purified 12 of these NBs and measured on-cell binding curves (Fig. 1, D to F, and fig. S1G). The three α_3NB_ binders exhibited the highest binding affinities, i.e., lowest dissociation constant (*K*_d_) values: 37 to 139 nM. α_2NB_ binder affinities were 205 to 2800 nM (Fig. 1F and table S2). Notably, it is not possible to correlate these affinities, which come from purified NBs at known concentrations, with the screening data binding scores (Fig. 1, B and C) obtained from unpurified NBs for which the concentration is only an estimate, between 1 to 12 μM (fig. S1A).

### Functionally silent and near-silent α_2_/α_3_ NBs do not bind intersubunit interfaces

**Fig. 2.**
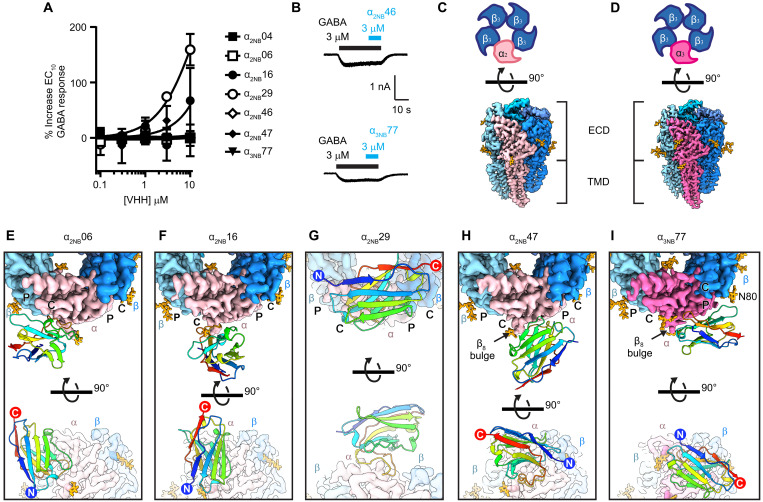
Functionally silent and near-silent α_2_/α_3_ NBs do not bind intersubunit interfaces. (**A**) NB modulation of whole-cell patch clamp EC_10_ GABA responses. Mean ± SEM (*n* = 3 to 6). (**B**) GABA (3 μM; ∼EC_10_) current responses before and after NB coapplication (top: α_2_β_3_γ_2_; bottom: α_3_β_3_γ_2_). (**C** and **D**) Top-down schematic view and cryo-EM map side view of (C) α_2_β_3_ and (D) α_3_β_3_ receptors containing a single α-subunit. (**E** to **I**) Top-down and side views of GABA_A_ receptor cryo-EM maps bound by ribbon representations of NBs (rainbow coloring; blue N terminus to red C terminus). Binding positions of (E) α_2NB_06, (F) α_2NB_16, (G) α_2NB_29, (H) α_2NB_47, and (I) α_3NB_77. Subunit ECD principal (P) and complementary (C) faces are indicated.

To examine NB functional effects, we recorded whole-cell patch-clamp currents from HEK293T cells expressing α_2_β_3_γ_2_ or α_3_β_3_γ_2_ GABA_A_ receptors. Seven NBs exhibited silent or near-silent effects on effective concentration (EC)_10_ GABA responses (Fig. 2, A and B) (α_2NB_05 and α_3NB_56 were not tested because of sequence similarity to α_2NB_04 and α_3NB_177, respectively). α_2NB_04, α_2NB_46, and α_2NB_47 from CDR3 α_2_-family 1 and α_2NB_06 and α_3NB_77 from CDR3 α_3_-family 1 were nonmodulatory. α_2NB_16 exhibited a weak 34 ± 30% PAM effect only at 10 μM, 30-fold above its *K*_d_ of 409 ± 188 nM, suggesting a requirement either to occupy all receptors to reveal any effect or a nonspecific effect. α_2NB_29 exhibited a low potency PAM effect only above 3 μM, fivefold above its *K*_d_ of 693 ± 117 nM.

To determine the binding modes for six of the seven (near-)silent NBs by cryo-EM (α_2NB_46 was not determined, but its CDR is similar to α_2NB_04 and α_2NB_47; fig. S1B), we used GABA_A_ receptors comprising one α_2_ or α_3_ subunit and four β_3_ subunits, due to high yield and the ability to bind a β-β megabody fiducial for tumbling in solution to avoid problems with preferential orientation (Fig. 2, C and D, and table S3) (*34*). The only exception was for α_2NB_04, which was solved bound to an α_2_β_3_γ_2_ heteromer (table S4). The GABA-bound β-α-β ECD region of the αβ (1:4) receptors was structurally highly similar to the equivalent region of GABA_A_ αβγ heteromers (*3*) (RMSD Cα α_2_β_3_ or α_3_β_3_ versus α_1_β_3_γ_2_ ECD < 0.9 Å for all structures) (fig. S2, A and B).

Five distinct epitopes were identified (Fig. 2, E to I, and fig. S2, C and D). α_2NB_06 binds to the outer face of the α_2_ subunit ECD, adjacent to the GABA pocket and close to but not contacting the principal (P) face of the β_3_ subunit (Fig. 2E and fig. S2, E and K). α_2NB_16 holds a broadly similar bound orientation to α_2NB_06, both NBs having the N- and C-terminal β strands perpendicular pointing away from the membrane plane, but the α_2NB_16 contact zone is shifted ∼2 Å away from the β-subunit (Fig. 2F and fig. S2, F and L). In contrast, α_2NB_29 binds above the α-subunit over its N-terminal α helix and its N- and C-terminal β strands are parallel to the membrane (Fig. 2G and figs. S2, G and M, and S3A). α_2NB_47 and related α_2NB_04 also have N and C termini approximately parallel to the membrane but instead bind to the outer face of the ECD, this time midway across, over the hypervariable β_8_ strand loop bulge (Fig. 2H and fig. S2, C, H, I, N, and O). α_3NB_77 also binds over the β_8_ strand loop bulge, but the N- and C-terminal orientation is flipped, and the contact zone is closer to the alternate β_3_ subunit complementary (C) face, but not contacting it (Fig. 2I and fig. S2, D, J, and P). The diverse binding modes of these NBs mean that between them, they cover almost the entire available space of the upper α-subunit ECD (fig. S2D). Given that receptor inhibition and activation are reliant on stabilizing specific intersubunit interface alignments, the fact that none of these NBs make contacts across the intersubunit interfaces to β-subunit residues is consistent with the weak or absence of binder-induced functional effects measured by electrophysiology.

### α_2NB_25 blocks GABA binding and inhibits hippocampal synaptic signaling

**Fig. 3.**
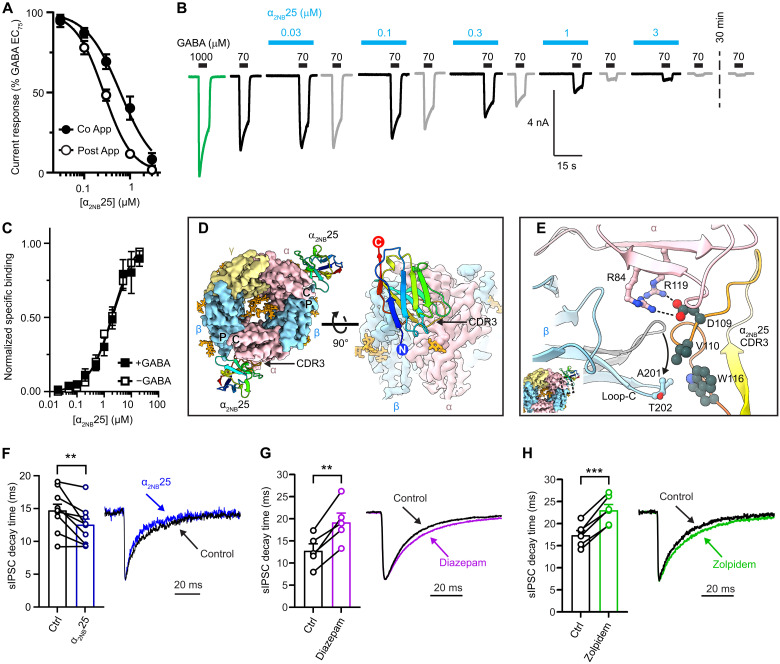
α_2NB_25 blocks GABA binding and inhibits synaptic signaling. (**A**) α_2NB_25 dose response curve for inhibition of EC_75_ GABA α_2_β_3_γ_2_ responses. Mean ± SEM (*n* = 3). Co App, coapplication of GABA with NB; Post App, postapplication of GABA after NB wash-off. (**B**) GABA current responses during and after the application of progressively increasing doses of α_2NB_25 (α_2_β_3_γ_2_). Green trace is *I*_max_. Gray traces are applications 2 min after the preceding black trace. (**C**) Specific binding curve for α_2NB_25 to α_2_β_3_ receptors in the presence versus absence of 500 μM GABA. Mean ± SEM (*n* = 3). (**D**) Top-down and side views of the α_2_β_3_γ_2_ cryo-EM map bound by α_2NB_25 (ribbon representation, rainbow coloring, blue N terminus to red C terminus). β-Subunit P and α-subunit FA (C) faces are indicated. (**E**) α_2NB_25 CDR3 bound across the α_2_/β_3_ interface. Electrostatic interactions shown as dashed lines. Nitrogen atoms: blue; oxygen atoms: red. A structurally aligned loop-C overlay from GABA-bound α_1_β_3_γ_2_ [Protein Data Bank (PDB): 6HUO] is shown in gray for comparison. Curved arrow indicates α_2NB_25 CDR3 forcing loop-C outward. (**F** to **H**) Histograms measuring spontaneous inhibitory postsynaptic current (sIPSC) amplitudes from dentate gyrus granule cells (DGGCs) before and after incubation with (F) 3 μM α_2NB_25, (G) 500 nM diazepam (DZP), and (H) 50 nM zolpidem. Insets show overlays of averaged sIPSCs before (black) and after (colors indicated) ligand incubations. Ctrl, control. Mean ± SEM (α_2NB_25 *n* = 8 cells, DZP *n* = 5 cells, zolpidem *n* = 6 cells). *P* values (two sided) were calculated by paired Student’s *t* test; **P < 0.01; ***P < 0.005.

Application of increasing concentrations of α_2NB_25 to α_2_β_3_γ_2_ receptors revealed a progressive and complete current blockade of EC_75_ GABA responses, with a median inhibitory concentration (IC_50_) = 0.53 μM (negative logarithm pIC_50_: 6.273 ± 0.103) (Fig. 3A). Inhibition sensitivity increased against subsequent EC_75_ GABA responses although the NB was absent from the postapplied solution; IC_50_ = 0.26 μM (pIC_50_: 6.580 ± 0.051), *P* < 0.029, paired *t* test, and GABA action could not be restored even 30 min later (Fig. 3, A and B). Thus, α_2NB_25 locks receptors in an inhibited state that subsequent GABA applications cannot compete off, suggesting a noncompetitive mode of action. This is further supported by α_2NB_25 being equally effective against low EC_15_ and high EC_75_ GABA doses (fig. S4, A and B). In addition, consistent with an inability of GABA to compete with α_2NB_25, its binding affinity for α_2_β_3_ receptors in the presence versus absence of GABA was identical (*K*_d_ = 2309 ± 778 nM versus 2391 ± 697 nM, respectively; *P* = 0.94, *n* = 3, unpaired *t* test) (Fig. 3C and table S2).

A 3.0-Å resolution cryo-EM structure revealed two copies of α_2NB_25 bound to the α_2_β_3_γ_2_ receptor, one over each GABA pocket β-α interface (Fig. 3D, fig. S4C, and table S4). Consistent with being α_2_ selective (table S1), α_2NB_25 mainly contacts the α-subunit (∼870 Å^2^ versus for the β-subunit ∼150 Å^2^) containing between 8 and 12 unique α_2_ residues versus each of the other five α-subunits (fig. S4D and table S5) and not being β-subtype selective (fig. S4E). Although the α_2NB_25 CDR3 sequence is unrelated to the silent binder α_2NB_06, its orientation is similar (NB framework RMSD C_α_ = 0.5 Å). A minor tilt angle variation means α_2NB_06 is further away from the β-subunit loop-C, ∼3.8 Å for α_2NB_06 versus ∼2.3 Å for α_2NB_25 (fig. S4F), and therefore unable to bridge the β-α interface and modulate function. In contrast, α_2NB_25 CDR3 Asp^109^ (D109) forms a double salt bridge with α_2_ Arg^84^ (R84) and Arg^119^ (R119) (3.9 and 3.2 Å, respectively) to stabilize the adjacent Val^110^ (V110) and Tryp^116^ (W116) residues within van der Waals (vdW) range of the β-subunit loop-C Ala^201^ (A201) and Thr^202^ (T202) to sterically block closure and GABA binding (Fig. 3E). Consistent with this, no GABA density is observed although it was included in the sample, and the receptor occupies an inhibited receptor conformation with closed 9′ channel gate (fig. S4, G to I) (*3*). CDRs 1 and 2 do not contact either subunit, but framework regions (FR) 1 and 2 contribute contacts (figs. S1B and S4J). A β-subunit Ala^201^→Tyr (A201Y) or Thr^202^→Tyr (T202Y) mutation to increase volume and create steric hindrance with α_2NB_25 did not affect binding (fig. S4K). Similarly, A201Y did not reduce inhibitory potency (fig. S4L), whereas T202Y precluded analysis due to minimal GABA responses even at a high 30 mM dose (28 ± 35 pA; *n* = 5). The lack of effect on Nb125 binding and modulation by these mutations can be explained by the flexible outward loop-C pose allowing Tyr rotamers to be accommodated and avoid steric clashes with α_2NB_25 (fig. S4, M and N). Thus, inhibition is independent of loop-C composition and only contingent on physically blocking closure. As has been previously observed for α-cobratoxin–inhibited α_1_β_3_ GABA_A_ receptors (*25*), α_2NB_25 does not directly overlap to compete with the bound GABA pose (fig. S4H). We propose that the forced outward orientation of the loop-C cap wedges α_2NB_25 between the β- and α-subunits to block dissociation and washout of inhibition during electrophysiological recordings. Overall, GABA and α_2NB_25 bind mutually exclusive conformations of the same pocket, but inhibition manifests as being noncompetitive, presumably due to an inability to wash-off the NB.

To evaluate its effect on physiological activity, we obtained electrophysiological recordings from dentate gyrus granule cells (DGGCs) in acute hippocampal brain slices, which contain a subpopulation of α_2_ containing receptors (*35*). While frequency, amplitude, and rise times of spontaneous inhibitory postsynaptic currents (sIPSCs) were unaffected (fig. S4, O to Q), the decay time was significantly shortened in the presence of 3 μM α_2NB_25 from 14.7 ± 0.9 to 12.6 ± 0.9 ms (*P* = 0.006, *n* = 9, *t* = 3.7, paired *t* test), thereby reducing GABAergic inhibitory drive (Fig. 3F). To rationalize the proportionately small effect, ∼15% versus the 100% inhibition achieved on a pure GABA_A_ α_2_-receptor population at 3 μM α_2Nb_25 (Fig. 3C), we assessed the α_1_-containing subtype synaptic contribution. Diazepam and zolpidem are similarly efficacious small-molecule PAMs; however, at 50 nM, zolpidem is α_1_ selective occupying 50% of this subtype, whereas at 500 nM, diazepam saturates 100% of α_1/2/3/5_-containing subtypes (*36*, *37*). Zolpidem (50 nM) extended IPSC decay time by 32% versus 50% for 500 nM diazepam (Fig. 3, G and H), implying that α_1_ is the major subtype in this neuronal population, with other subtypes comprising smaller subpopulations. This is consistent with α_2_Nb25 achieving strong inhibition of the minor α_2_ subpopulation to overall achieve 15% inhibition of IPSCs. In contrast, α_2NB_25 did not affect the GABAergic tonic current size or root mean square (RMS) noise (fig. S4, R and S), consistent with the idea that other GABA_A_R subtypes may underpin basal tonic current levels in these neurons (*38*).

### α_2NB_00 stabilizes GABA binding and increases hippocampal synaptic and tonic signaling

**Fig. 4.**
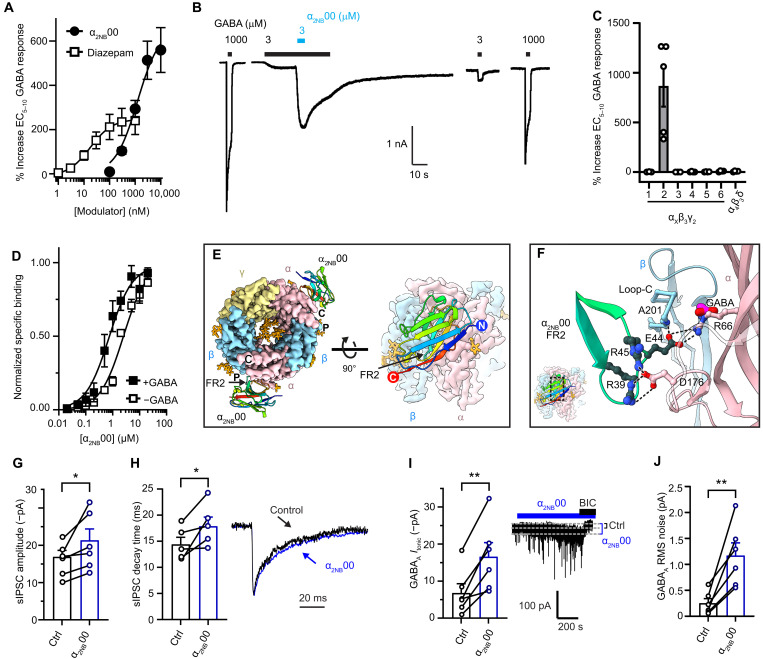
α_2NB_00 stabilizes GABA binding and increases hippocampal synaptic and tonic signaling. (**A**) α_2NB_00 and DZP dose response curves for potentiation of EC_5–10_ GABA responses on recombinant α_2_β_3_γ_2_. Mean ± SEM (*n* = 5 and 6). (**B**) GABA current responses during and after coapplication of 3 μM α_2NB_00 α_2NB_00 on recombinant α_2_β_3_γ_2_. (**C**) Histogram showing potentiation by 3 μM α_2NB_00 at α_1_-α_6_ subunit containing α_X_β_3_γ_2_ receptors. Mean ± SEM (*n* = 3). (**D**) Specific binding curve for α_2NB_00 to α_2_β_3_ receptors in the presence versus absence of 500 μM GABA. Mean ± SEM (*n* = 3). (**E**) Top-down and side views of the α_2_β_3_γ_2_ cryo-EM map bound by α_2NB_00 (ribbon representation, rainbow coloring, blue N terminus to red C terminus). β-subunit P and α-subunit C faces are indicated. (**F**) α_2NB_00 FR2 bound across the α_2_/β_3_ interface; β_3_ loop-C shown as Cα-backbone representation. Electrostatic interactions and H bonds shown as dashed lines. Nitrogen atoms: blue; oxygen atoms: red; GABA: magenta. (**G** to **J**) Histograms measured from DGGCs before and after incubation with 300 nM α_2NB_00, for (G) sIPSC amplitudes, (H) sIPSC decay times, (I) tonic current, and (J) RMS tonic noise. Inset for (H) shows overlays of averaged sIPSCs before (black) and after (blue) α_2NB_00 incubation. Inset for (I) shows tonic current before and after incubation with α_2NB_00 and then the pan GABA_A_ receptor antagonist, bicuculline (BIC). Data are mean ± SEM (*n* = 6 cells). *P* values (two sided) were calculated by paired Student’s *t* test; *P < 0.05; **P < 0.01.

Application of α_2NB_00 to α_2_β_3_γ_2_ receptors revealed that EC_5–10_ GABA responses were efficaciously potentiated by 760 ± 130% (EC_50_ = 1.2 ± 0.2 μM, *n* = 6) versus 240 ± 70% for diazepam (Fig. 4, A and B). Close to saturating 3 μM doses slowed the wash-off of GABA responses, creating a prolonged leak current, continued potentiation of EC_5–10_ responses, and rundown of the maximal response presumably due to desensitization (Fig. 4B and fig. S5, A and B). Despite the massive potentiation, 3 μM α_2NB_00 PAM remains completely α_2_-containing subtype selective (Fig. 4C). α_2NB_00 affinity for α_2_β_3_ receptors revealed a higher affinity for the GABA-bound conformation, consistent with its PAM action, although this did not reach significance (*K*_d_ = 892 ± 275 nM versus 1814 ± 751 nM, respectively; *P* = 0.31, *n* = 3, unpaired *t* test) (Fig. 4D and table S2).

As was the case for the α_2NB_25 inhibitor, a 2.7-Å resolution structure revealed two copies of α_2NB_00 bound to each GABA pocket (Fig. 4E, fig. S3, B and C, and table S4). Similarly, α_2NB_00 forms a larger contact zone with the α-subunit than the β-subunit (∼800 Å^2^ versus ∼140 Å^2^), making between 10 and 11 unique α_2_ putative residue contacts versus each of the other α-subunits, explaining its high α_2_-containing subtype selectivity (Fig. 4C and fig. S5D), whereas α_2NB_00 is not β-subtype selective (fig. S5, E and F, and table S5B). α_2NB_00 β strands and N and C termini are rotated clockwise by ∼90° versus α_2NB_25 (Figs. 3D vs. 4E), meaning CDR3 contacts are focused on the α-subunit (fig. S5G) and FR2 forms the β-α intersubunit contacts (Fig. 4F). Again, salt bridges form the key contacts for α_2NB_00. FR2 Arg^39^ (R39) and Arg^45^ (R45) double salt bridge to α_2_ Asp^76^ (D176) (2.2 and 3.3 Å, respectively) (Fig. 4F), whereas FR2 Glu^44^ (E44) directly connects the β-α interface by salt bridging with α_2_ Arg^66^ (R66) (3.7 Å) to hold it in the GABA binding conformation and H-bonding the β-subunit loop-C A201 backbone amide (2.2 Å) to stabilize it in an inward loop-C conformation for coordination with GABA (Fig. 4F). Overall, the double stabilization traps bound GABA in its pocket, explaining the slow wash-off observed for GABA responses during whole-cell recordings. In contrast to α_2NB_25 where an A201Y or T202Y mutation did not affect binding, for α_2NB_00 binding was almost ablated (fig. S5H). This is due to the PAM effect closing loop-C leaving no space for Tyr rotamers to be accommodated (fig. S5, I and J). The 9′ activation gate is open, but the −2′ desensitization gate is closed, matching the α_2_β_3_γ_2_ receptor bound by GABA and silent α_2NB_04 and GABA-bound α_1_-containing receptors (fig. S5, K to L) (*3*). Consistent with its modulation effect stemming from potentiation, not direct activation, application of 3 μM α_2NB_00 in the absence of GABA induced very low levels of direct activation: 1.9 ± 0.7% of GABA *I*_max_ (fig. S5M). Prolonged 5-min low level activation coincided with an ∼20% reduction in the GABA *I*_max_, possibly due to the accumulation of desensitized receptor states (fig. S5N).

The application of 300 nM α_2NB_00 to DGGCs significantly increased the amplitude and decay time of sIPSCs: amplitudes: 16.9 ± 1.7 to 21.4 ± 3.0 pA, *P* = 0.025, *n* = 6, *t* = 2.586; decay times: 14.4 ± 1.4 to 17.9 ± 1.8 ms, *P* = 0.029, *n* = 5, *t* = 2.635, both paired *t* tests (Fig. 4, G and H); but did not affect frequency or rise time (fig. S5, O and P). Despite α_2_ being a minor subpopulation and α_2Nb_00 being applied at only an EC_15_ dose (300 nM; Fig. 4A), decay time was extended by 26 ± 12%, a comparable effect to that of the clinical PAM, zolpidem, at a dose selective for the major α_1_ subpopulation (33 ± 4%; Fig. 3H). In contrast to the α_2NB_25 inhibitor, which did not affect tonic leak, α_2NB_00 did detectably increase this current, from 6.8 ± 2.5 to 16.6 ± 3.8 pA (*P* = 0.002, *n* = 6, *t* = 5.024), and associated GABA_A_R-mediated tonic RMS noise: 0.25 ± 0.09 to 1.17 ± 0.25 pA (*P* = 0.0036, *n* = 6, *t* = 4.383, both paired *t* tests; Fig. 4, I and J). Increased leak likely results from an α_2NB_00 PAM effect on both synaptic and extrasynaptic α_2_-containing receptors to enhance their sensitivity such that they can detect and respond to low GABA levels in the tissue milieu, something indirectly demonstrated using nonselective neurosteroids (*35*).

### α_2_ inhibitor, silent, and PAM NBs exhibit distinct effects on cortical network activity

**Fig. 5.**
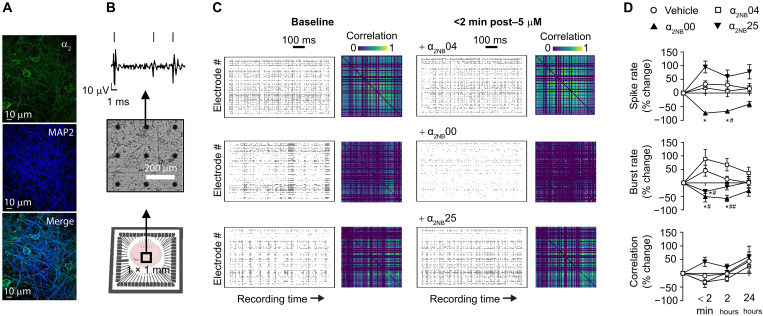
α_2_ inhibitor, silent, and PAM NBs exhibit distinct effects on cortical network activity. (**A**) Immunofluorescence image of DIV14 cortical culture GABA_A_ α_2_ subunit (green) and dendritic marker Microtubule-Associated Protein (MAP)2 (blue). (**B**) Top: Action potentials (spikes) from one MEA electrode – spike times indicated by black vertical lines. Middle: Electrode that recorded the signal on a representative MEA grid zoomed into 9 of the 60 electrodes. Bottom: Full grid schematic. (**C**) Representative baseline network activity (left) versus 2-min acute exposure (right). Each electrode contributes one row of recording to the black and white grid. This is subsequently paired via colored raster plot to show spike time correlation with every other electrode for analyzing burst firing. (**D**) Summary of NB effects. Mean ± SEM (*n* = 4 to 8). *P* values calculated by nonparametric one-way analysis of variance (ANOVA) Kruskal-Wallis test. *P < 0.05; versus silent α_2NB_04 binder control. ^#^*P* < 0.05; ^##^*P* < 0.01; versus vehicle control.

The ability to change GABAergic signaling in individual neurons predicts effects on wider network activity. To explore this, we tested α_2_ NBs on 14 days in vitro (DIV) cortical neuronal cultures grown on multielectrode arrays (MEAs) to measure action potential firing and burst synchronization. Cultures were confirmed to express the GABA_A_ receptor α_2_ subunit by immunofluorescence (Fig. 5A). α_2NB_04, the silent modulator, did not alter spike rate firing versus vehicle control (Fig. 5, C and D). Consistent with a PAM effect on inhibitory signaling, 5 μM α_2NB_00 reduced network excitability, i.e., the firing (spike) rate, at 2-min and 2-hour time points, by 74 ± 5 and 67 ± 7%, *n* = 6 respectively below baseline, and significantly below vehicle and silent α_2NB_04 controls (Fig. 5, B to D). However, this effect was no longer significant after 24 hours suggestive of GABAergic desensitization due to the high dose applied or NB degradation. In contrast, α_2NB_25 inhibitor showed a visible trend in the reverse direction toward increased firing rate immediately after application, consistent with reducing inhibitory signaling to boost excitation, although the effect was not significant (Fig. 5, C and D).

α_2NB_04 did not reduce the burst firing rate nor alter it relative to the vehicle control (Fig. 5D). However, the α_2NB_00 PAM did significantly reduced the synchrony of burst firing across the neuronal network, i.e., reduced how often neurons coordinate firing, again both immediately after application and at the 2-hour timepoint by 51 ± 22 and 58 ± 13% (*n* = 6; Fig. 5D). The α_2NB_25 inhibitor did reach significance of effect for reducing phasic burst activity immediately after application by 32 ± 8% (*n* = 6; Fig. 5D).

### α_3NB_83 induces a GABA-activated ECD and increases hippocampal synaptic signaling

**Fig. 6.**
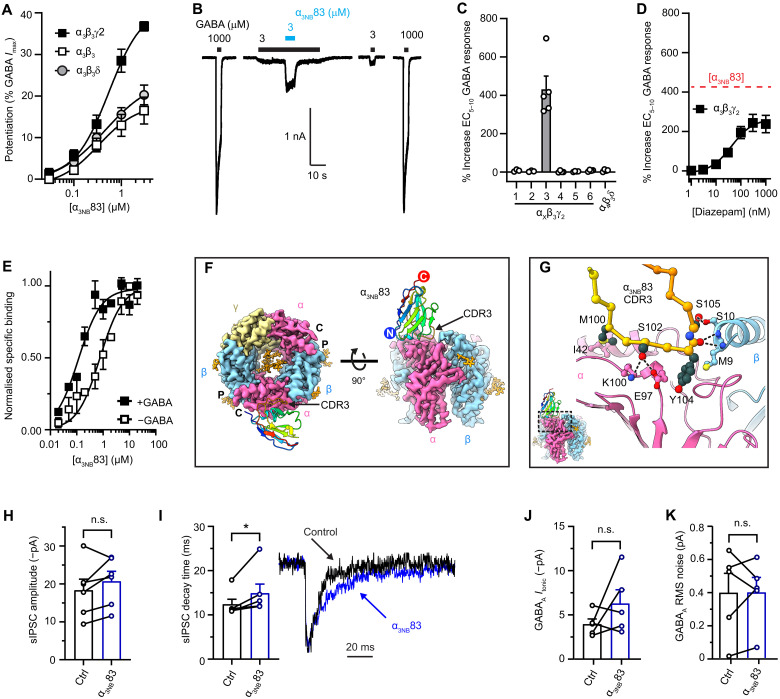
α_3NB_83 encourages β-subunit tilting and increases hippocampal synaptic signaling. (**A**) α_3NB_83 dose response curves for potentiation of EC_5–10_ GABA responses, shown as percentage GABA *I*_max_. Mean ± SEM (*n* = 3). (**B**) GABA current responses on α_3_β_3_γ_2_ during and after coapplication of 3 μM α_3NB_83. (**C**) Histogram showing potentiation by 3 μM α_3NB_83 at α_1_-α_6_ subunit containing α_X_β_3_γ_2_ receptors. Mean ± SEM (*n* = 3). (**D**) Diazepam dose response curve for potentiation of EC_10_ GABA responses. Mean ± SEM (*n* = 6). (**E**) Specific binding curve for α_3NB_83 to α_3_β_3_ receptors in the presence versus absence of 500 μM GABA. Mean ± SEM (*n* = 3). (**F**) Top-down and side views of the α_2_β_3_γ_2_ cryo-EM map bound by α_3NB_83 (ribbon representation, rainbow coloring, blue N terminus to red C terminus). β-subunit P and α-subunit C faces are indicated. (**G**) α_3NB_83 CDR3 (Cα-backbone representation) bound across the α_3_/β_3_ interface. Putative H bonds shown as dashed lines. Nitrogen atoms: blue; oxygen atoms: red; sulfur: yellow. (**H** to **K**) Histograms measured from DGGCs before and after incubation with 3 μM α_3NB_83, for (H) sIPSC amplitudes, (I) sIPSC decay times, (J) tonic current, and (K) RMS tonic noise. Inset for (I) shows overlays of averaged sIPSCs before (black) and after (blue) incubation with α_3NB_83. Mean ± SEM (*n* = 5 cells). *P* values (two sided) were calculated by paired Student’s *t* test except for sIPSC decay times (Wilcoxon matched-pairs signed-rank test); n.s. (not significant), *P* > 0.05; *P < 0.05.

Coapplication of α_3NB_83 to α_3_β_3_γ_2_ receptors increased EC_5–10_ GABA responses by 432 ± 69% (EC_50_ = 0.59 μM, pEC_50_ = 6.226 ± 0.128) versus 240 ± 50% for diazepam (Fig. 6, A to D). Unlike for α_2NB_00, 3 μM doses of α_3NB_83 did not visibly slow GABA_A_ receptor inactivation nor was there residual potentiation of subsequent EC_5_ responses or rundown of maximal GABA responses (Fig. 6B and fig. S5, A and B). Similar to α_2NB_00, α_3NB_83 is completely α_3_-containing subtype selective at a 3 μM dose (Fig. 6C). As is expected for a PAM, α_3NB_83 exhibits a significantly higher affinity for the GABA-bound conformation (*K*_d_ = 139 ± 25 nM versus 747 ± 191 nM, respectively; *P* = 0.035, *n* = 3, unpaired *t* test) (Fig. 6E and table S2).

To fully understand the mechanism of action of α_3NB_83, we solved four structures of the α_3_β_3_γ_2_ receptor in the range 2.7- to 3.2-Å resolution: bicuculline inhibitor + silent α_3NB_77; GABA + silent α_3NB_77; GABA preincubated before addition of α_3NB_83 (GABA_PRE_ + α_3NB_83); and α_3NB_83 preincubated before addition of GABA (α_3NB_83_PRE_ + GABA) (table S4). Both cryo-EM structures of α_3_β_3_γ_2_ + GABA + α_3NB_83 show a single NB bound, on top of the single α-β interface (Fig. 6F and fig. S6, A and B). This interface has previously been targeted for modulation by nonselective and α_6_-selective small-molecule PAMs (*39*). α_3NB_83 interacts almost exclusively with the α-subunit rather than the β-subunit (∼620 Å^2^ versus ∼110 Å^2^) (Fig. 6G), with α-selectivity conveyed by between two and four unique α_3_ residue contacts versus each of the other α-subunits (fig. S6C and table S5C), whereas β_2_ versus β_3_ selectivity is lacking (fig. S5E) and not expected because the NB contact residues in the β-subunit, Met^9^ (M9) and Ser^10^ (S10), are conserved across β_1–3_. Nevertheless, the β-subunit contacts are important because NB density is almost entirely absent at the alternative α_3_-γ_2_ interface (fig. S6B). While CDR1 and 2 provide some contacts (fig. S6D), the main interaction zone comes from CDR3 residues 100 to 105, which run flat along the top of the α_3_ subunit. Met^100^ (M100) forms putative contacts to α_3_ Ile^42^ (I42), and Ser^102^ (S102) forms a putative H bond to α_3_ Asp^97^/Lys^100^ (D97/K100) (3.9 and 4.1 Å, respectively) (Fig. 6G). With the β-subunit, the Tyr^104^ (Y104) phenyl ring forms putative contacts with the M9 side chain, and Ser^105^ (S105) forms a putative H bond with the S10 side chains (2.3 Å). In addition, the Y104 backbone carbonyl oxygen makes putative H-bond interactions with the M9 and S10 backbone amides (∼3.6 Å) (Fig. 6G). An Met^9^→Glu (M9E) or Ser^10^→Gly (S10G) mutation to the γ_2_ equivalents did not reduce binding affinity, but Met^9^→Pro (M9P) to disrupt the putative NB-β-subunit backbone interaction significantly reduced affinity threefold (fig. S6, E to G). Thus, β-subunit residue identity and the M9 backbone modestly affect α_3NB_83 binding, consistent with an interaction, albeit the larger α_2_-subunit contact zone is the main binding determinant. Although the binding assay confirms surface expression of α_3_β_3_^M9P^γ_2_ receptors and the α-β interface, which binds α_3NB_83, the receptor was nonresponsive to GABA (*n* = 11 cells tested), suggesting that this interface can indeed affect GABA transduction, consistent with the ability of α_3NB_83 to also impact (enhance) transduction.

Comparison of the four structures reveals that GABA binding induces the classical β-subunit tilt and loop-C closure (fig. S6, H and I) (*3*, *4*, *25*). Both α_2NB_83-bound structures match the α_3_β_3_γ_2_ + GABA + silent α_2NB_77 conformation (global RMSD versus GABA_PRE_ + α_3NB_83 = 0.23 Å; versus α_3NB_83_PRE_ + GABA = 0.46 Å), showing that α_2NB_83 does not induce further changes to this conformation. However, uniquely for the structure preincubated with α_3NB_83 before GABA, the density for GABA is missing from the β-subunit contacting α_3NB_83 at a map contour level giving strong neurotransmitter density in the other pocket (fig. S6, J and K). Thus, in the absence of GABA, α_3NB_83 binds and pushes the β-subunit via its the N-terminal α helix to induce ECD tilting and loop-C closure of the neurotransmitter pocket, mimicking GABA, and this prevents GABA that is subsequently added from accessing the shut pocket. This can also explain why α_3NB_83 does not slow inactivation versus α_2NB_00 because it cannot trap GABA at the second pocket. The PAM effect of α_3NB_83 also extends to α_3_β_3_ and α_3_β_3_δ receptors, supporting the existence of the α-β interface in these pentamers (Fig. 6A and fig. S6, L and M) (*25*, *40*). In contrast with the α_2_β_3_γ_2_ receptor, which exhibits an open 9′ activation gate in response to GABA binding (fig. S5K), the α_3_β_3_γ_2_ gate is closed in all four structures (fig. S6N). This suggests that the energetics of transmission from ECD to TMD is lower for the α_3_-containing subtype in the nanodisc preparation, as has been reported for alternative GABA_A_R isoforms (*25*).

Application of 3 μM α_3NB_83 to DGGCs did not affect sIPSC frequency, amplitude, or rise time but did exert a PAM effect to significantly increase decay time from 12.4 ± 1.1 to 14.9 ± 2.1 ms (*P* = 0.031, *n* = 6, *W* = 21, Wilcoxon matched-pairs signed-rank test; Fig. 6, H and I, fig. S6, O and P). At this close-to saturating dose, decay time was extended by 18 ± 6%, less than that achieved by the α_1_-selective zolpidem dose (33 ± 4%; Fig. 3H), demonstrating that α_3_ contributes to synaptic responses but is a minor subpopulation, as was shown earlier for α_2_. In contrast to α_2NB_00, α_3NB_83 did not, however, affect tonic leak (4.0 ± 0.6 to 6.3 ± 1.5 pA, *P* = 0.23, *n* = 5, *t* = 1.404, paired *t* test; Fig. 6, J and K), suggesting that α_3_-containing receptors have a specialized phasic signaling role in these neurons.

### α_2NB_00 and α_3NB_83 differentially boost GABAergic amygdala signaling

**Fig. 7.**
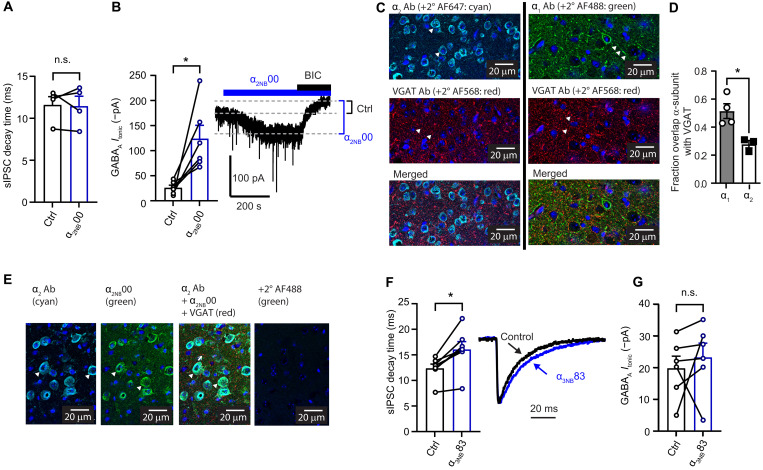
α_2NB_00 and α_3NB_83 differentially boost GABAergic amygdala signaling. (**A** and **B**) Histograms measured from BLA principal neurons before and after incubation with 3 μM α_2NB_00 for (A) sIPSC decay or (B) tonic current. Mean ± SEM (*n* = 4 to 6 cells). *P* values (two sided) were calculated by paired Student’s *t* test; n.s., *P* > 0.05; *P < 0.05. Inset for (B) shows tonic current before and after incubation with α_2NB_00 and then bicuculline (BIC). (**C**) BLA brain slice immunofluorescence for GABA_A_ α_2_ subunits (represented in cyan; left) or α_1_ subunits (green; right) versus VGAT localization (red). α_2_ is diffuse somatic, α_1_ is spread along projections, and VGAT is punctate (examples indicated by white arrowheads). (**D**) Histogram of α_1_ versus α_2_ colocalization with VGAT. Mean ± SEM (*n* = 4 to 6 cells). *P* values (two sided) were calculated by paired Student’s *t* test; n.s., *P* > 0.05; *P < 0.05. (**E**) GABA_A_ α_2_ immunofluorescence stained by anti-GABA_A_ α_2_ commercial antibody versus α_2NB_00 fused to human immunoglobulin G (IgG), both showing somatic localization (examples indicated by white arrowheads; VGAT: open arrow), which is absent in antihuman secondary alone negative control. (**F** and **G**) Histograms measured from BLA principal neurons before and after incubation with 3 μM α_3NB_83 for (F) sIPSC decay or (G) tonic current. Mean ± SEM (*n* = 4 to 6 cells). *P* values (two sided) were calculated by Wilcoxon matched-pairs signed-rank test; n.s., *P* > 0.05; *P < 0.05. Inset for (F) shows overlays of averaged sIPSCs before (black) and after (blue) incubation with α_3NB_83.

Nonselective GABA_A_ receptor PAMs such as diazepam are taken clinically to treat anxiety (*41*). Genetic and pharmacological studies have shown that the α_2_ subunit is an important contributor to the anxiolysis, while α_3_ subunit involvement is less clear (*7*–*9*, *12*). The amygdala is involved in fear processing, anxiety (*42*), and their related disorders (*43*) and expresses α_2_ and α_3_ subunits, which are likely contributors to the anxiolytic effects of PAMs (*44*, *45*). To causally explore this further with our efficacious and selective NB tools, we tested 3 μM doses of the α_2NB_00 and α_3NB_83 PAMs for impacts on principal neuron GABAergic signaling in acute basolateral amygdala (BLA) brain slices. α_2NB_00 did not affect sIPSC properties, suggesting that any contribution of α_2_-containing GABA_A_Rs to phasic signaling is too small to detect in these BLA principal neurons (Fig. 7A, and fig. S7, A to C). However, α_2NB_00 did significantly increase tonic leak (26.0 ± 5.1 to 123.8 ± 26.7 pA; *P* = 0.02, *n* = 6, *t* = 3.365, paired *t* test), demonstrating the presence of extrasynaptic α_2_-containing receptors (Fig. 7B). Consistent with this, immunohistochemistry revealed that α_2_-subunit fluorescence was predominantly somatic, with 72 ± 2% not being colocalized with the synaptic marker vesicular GABA transporter (VGAT) (Fig. 7, C and D). This is significantly lower than for α_1_, 49 ± 7% (*P* < 0.05), which was localized to neuronal projections. Furthermore, α_2NB_00 showed the same somatic staining as the α_2_ antibody, indicating targeting of the same α_2_ population (Fig. 7E). Application of α_3NB_83 also potentiated GABAergic drive but did so via significantly increasing synaptic decay time by ∼30% (12.4 ± 0.9 to 16.1 ± 1.5 ms, *P* = 0.016, *n* = 7, *W* = 28, Wilcoxon matched-pairs signed-rank test), with no effect on tonic current (Fig. 7, F and G, and fig. S7, D to F), demonstrating a contribution from α_3_-containing receptors to phasic signaling.

### α_2NB_00 reduces anxiety-like behavior in rats in the EPM

**Fig. 8.**
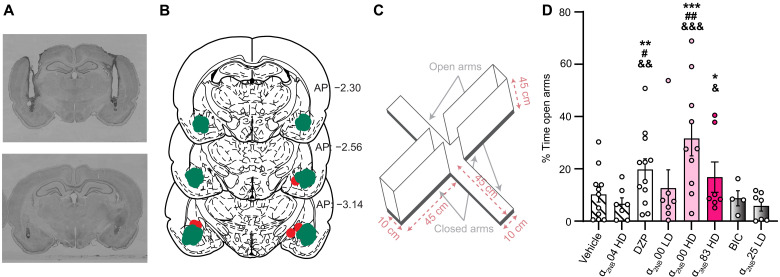
α_2NB_00 reduces anxiety-like behavior in rats in the EPM. (**A**) Representative cresyl violet–stained 35 μM brain sections containing cannula tracts (top) or the infusion site (bottom), which accurately targeted the BLA. (**B**) Cannula placements mapped onto coronal outlines from the Rat Brain Atlas. Accurate placement: green dots; misplacement: red dots (these rats were excluded from the behavioral analysis). (**C**) Schematic of the arms of the EPM in which the rats are placed for behavioral testing. (**D**) Histogram showing percentage time spent in the open arms (OA) of the EPM. Compared to controls (univariate ANOVA main effect of dose: *F*_6,58_ = 3.625, *P* = 0.004), intra-BLA infusions of DZP (14 mM), α_2NB_00 HD (high dose; 250 μM). and α_3NB_83 HD (250 μM) increased the percentage of time spent in the OA, indicative of reduced anxiety-like behavior. Mean ± SEM (*n* = 4 to 12 per treatment group). *P < 0.05; **P < 0.01; ***P < 0.001; planned comparisons versus pooled controls (vehicle and silent α_2NB_04 binder control combined). ^#^*P* < 0.05; ^##^*P* < 0.01; planned comparisons versus vehicle control only. ^&^*P* < 0.05; ^&&^*P* < 0.01; ^&&&^*P* < 0.001; planned comparisons versus silent α_2NB_04 binder control. LD (low dose; 40 μM); BIC (2.7 mM).

We next characterized NB modulation of BLA physiology in behaving animals by assessing the effect of direct intra-BLA infusions on anxiety-like behavior in rats in the EPM, an established assay to measure anxiety and the anxiolytic properties of drugs (Fig. 8, A to C) (*32*, *46*). Infusion of 0.5 μl per side at 0.3 μl/min, of 250 μM of a negative control NB—the silent binder α_2NB_04—had no more effect on the percentage of time spent exploring the open arms of the maze than a buffer solution negative control (Fig. 8D). However, 14 mM (*44*) diazepam or 250 μM of the PAM α_2NB_00 resulted in a clear anxiolytic effect as revealed by a significant increase in the percentage of time spent by the rats exploring the open arms, with α_2NB_00 achieving a greater anxiolysis than diazepam (main effect of drug: *F*_6,58_ = 3.625, *P* < 0.005; 19.74 ± 4.08 versus 8.81 ± 1.99, *P* < 0.010; and 31.66 ± 6.68 versus 8.81 ± 1.99, *P* < 0.001, respectively). α_2NB_00 also reached significance compared to all controls (*P* < 0.01) for increasing distance traveled in open arms, number of open arm entries, and total time in open arms, and for reducing time in the closed arms, whereas diazepam did not reach significance for increasing the number of open arm entries (*P* = 0.07) or for reducing time in the closed arms (*P* = 0.07) (fig. S8, G to J). In contrast, a lower dose of 40 μM α_2NB_00 was not anxiolytic (Fig. 8D). α_3NB_83 exhibited some evidence of anxiolytic activity, significantly increasing the percentage of time spent by the rats in the open arms compared to the silent α_2NB_04 control, 16.83 ± 5.81 to 6.61 ± 2.28 (*P* < 0.05), but not versus the buffer control, 16.83 ± 5.81 to 10.21 ± 2.93 (*P* > 0.05; Fig. 8D). Total distance traveled was not significantly reduced between any treatment group, suggesting an absence of sedative or motor coordination effects (fig. S8K). Overall, our combined acute slice electrophysiology and behavioral data show that GABA_A_ receptor NBs can differentially increase tonic and phasic signaling in the amygdala and exert anxiolytic effects.

## DISCUSSION

Here, we screened and characterized NBs against the GABA_A_ receptor α_2_- and α_3_-containing subtypes. Cryo-EM structures showed that functionally silent or near-silent binders occupy several distinct locations that together cover most of the upper accessible region of the α-subunit ECD. These binders cannot bridge the intersubunit interface to affect GABA-driven subunit realignment, explaining their (near-)silent action. By comparison, the three NB modulators all bound at locations bridging an intersubunit interface, enabling them to impact the energetics of GABA-driven transitions. Both the inhibitor α_2NB_25 and PAM α_2NB_00 bound over the GABA pocket with definable impacts to explain molecular mechanisms of action. However, the α_3NB_83 PAM bound the alternative α-β interface, which does not have a GABA binding pocket. Instead, in the absence of bound GABA, α_3NB_83 stabilized an ECD that mimicked the GABA-bound conformation by tilting the contacting β-subunit and closing loop-C, explaining its PAM effect, to act as a surrogate GABA molecule but via an entirely allosteric process. Previously, the small-molecule PAM, NS9283, has also been proposed as a surrogate agonist acting at a nonorthosteric ECD intersubunit pocket of the nicotinic acetylcholine receptor (*47*).

Screening across all NBs revealed almost complete α_2_- or α_3_-containing subtype selectivity in the cell-binding assay. This was corroborated functionally for the three modulators, which all bind epitopes in possession of up to 12 unique α-subtype–specific residues, accounting for the high selectivity by binding. This contrasts with small molecules, for which the high degree of residue conservation in the BZD modulator pocket has hampered attempts to obtain α-selectivity by binding, with the exception of the α_5_-containing subtype (*34*). Furthermore, while small-molecule α_2_/α_3_ selectivity has relied on reducing PAM strength to only 10 to 50% above an EC_3–10_ GABA dose to achieve efficacy-based selectivity (*48*), the NBs do not suffer from this limitation, producing strong modulation of 300 to 900%. This is also true for inhibition. α-Selective small-molecule inhibitors have only been demonstrated for the α_5_-containing subtype and only achieved up to ∼50% inhibition, which could account for the lack of efficacy in clinical trials (*34*). In contrast, α_2NB_25 achieves α_2_-selective shutdown that approaches 100%.

Testing in physiological systems demonstrates that NB modulators engage receptors to modulate synaptic and extrasynaptic signaling, network excitability, and animal behavior. The combination of high efficacy and selectivity resolves subtype contributions to the neuronal calculation with confidence, something that is much more challenging with small molecules (*49*). While the impacts on IPSCs were not as strong as for pure recombinant α_2_ and α_3_ receptor populations, this was expected on the basis of α_2_ and α_3_ being minor subpopulations in neurons. Despite this, effect sizes were comparable to small molecules acting on the major α_1_ population. Thus, high efficacy meant these NBs could reveal contributions from minor subpopulations, confirming utility for dissecting subtype involvements. The NBs dissected distinct extrasynaptic α_2_ tonic current versus synaptic α_3_ phasic IPSCs, suggesting that for the α_2_-containing subtype, potentiation of extrasynaptic receptors may be the cellular mechanism specifically contributing to reducing anxiety. In contrast, for the α_3_-containing subtype, phasic receptors are inferred, albeit the anxiolysis effects are weaker, and further studies will be required to fully establish a role. Previously, a genetic knock-in study of GABA_A_ receptor α_1_, α_2_ and α_3_ subunits with ablated diazepam sensitivity identified a diazepam-sensitive synaptic inhibition component in the BLA for α_2_ and α_3_ subtypes (*50*). However, this involved evoked IPSCs, which induce greater release of GABA and potential spillover effects that recruit extrasynaptic receptors, and also tonic inhibition was not investigated, so a direct comparison cannot be made to the NB modulation results described here.

Overall, we demonstrate here that NBs break the pharmacological deadlock on GABA_A_Rs that has stood for decades, offering high selectivity and high efficacy, a “have your cake and eat it” α-containing subtype modulation. We also show that these tools are effective in the modulation of synaptic and extrasynaptic targets in acute brain slices, modulate neuronal network firing patterns, and control in vivo anxiety in behaving mammals. Notably, antibodies cannot naturally cross the blood-brain barrier and can trigger immunogenic and cytotoxic effects, meaning these agents will likely require engineering and fusion to recently developed and optimized low-toxicity antibody brain delivery shuttles (*51*), for follow-on systemic studies and therapeutic development. We anticipate that these tools and future generations will substantially enhance GABAergic pharmacology and neuroscience and facilitate the development of alternative therapeutic modalities for the treatment of neuropsychiatric disorders.

### Limitations of the study

We determined our structures in lipid nanodiscs, and it is conceivable that the protein energetics and conformations might vary in comparison to those in cell membranes, although the cryo-EM structural data align with the binding and electrophysiological data for receptors in cell membranes. Some of the experiments had statistical analysis performed on *n* = 3 or 4, which reduces the power of the comparison. Potentiation by different modulators for direct comparison in the same cell could not be performed because of concerns about complete washout and drug interactions affecting interpretation. An inability to control the local drug concentration subsequent to in vivo administration into the amygdala along with limitations in tissue penetration and rapid diffusion from site of injection means that higher doses of diazepam and NBs were required to elicit effects than is required in vitro, and it is not possible to know the actual local concentration exerting the behavioral effect. This is consistent with previous approaches applying high doses of diazepam in anxiety and nociception animal behavior studies (*11*, *35*). The GABA_A_R α_3_ subunit is expressed on the X chromosome; however, only male rats were used in the EPM study. In addition, direct visualization of NB engagement with GABA_A_ receptors in the amygdala was not possible due to withdrawal of the cannula, causing local tissue damage combined with autofluorescence, clearance before cardiac perfusion, and unsuitability of the purification His tag for staining.

## MATERIALS AND METHODS

### GABA_A_ receptor construct design

Human GABA_A_ receptor α_1_/α_2_/α_3_/α_4_/α_5_/α_6_/β_3_/γ_2_/δ subunit wild-type mature protein sequences were used for recombinant electrophysiology experiments in this study (α_1_ UniProt P14867 entry Gln28 is Gln1 1 to 429 QPSL…PTPHQ; α_2_ UniProt P47869 entry Asn29 is Asn1 1 to 423 NIQE…LGVSP; α_3_ UniProt P34903 entry Gln29 is Gln1 1 to 464 QGES…MIRKQ; α_4_ UniProt P48169 entry Gln36 is Gln1 1 to 519 QNQK…SESLM; α_5_ UniProt P31644 entry Gln32 is Gln1 1 to 431 QMPT…AASPK; α_6_ UniProt Q16445 entry Lys20 is Lys1 1 to 434 KLEV…SSSVE; β_3_ UniProt P28472 isoform 1 entry Gln26 is Gln1 1 to 448 QSVN…LYYVN; γ_2_ UniProt P18507 isoform 1 entry Gln40 is Gln1 1 to 428 QKSDD…SYLYL; δ UniProt O14764 entry Met25 is Met1 1 to 428 MNDI…AAYAM). To boost expression and purification yields for NB library generation, cell binding studies, and cryo-EM, the constructs were modified to substitute the M3-M4 ICD with either a glvi linker sequence SQPARAA or a modified glvi sequence containing the *Escherichia coli* soluble cytochrome B562RIL41 (BRIL; amino acids 23 to 130, ADLE…QKYL, UniProt P0ABE7) to give the sequence SQPAGT-BRIL-TGRAA (*23*). Regions substituted were as follows: α_1_ Arg^313^-Ser^390^; α_2_ Arg^312^-Ser^387^; α_3_ Arg^337^-Ser^425^; α_4_ Ile^311^-Ser^481^; α_5_ Arg^316^-Ser^392^; α_6_ Leu^311^-Ser^396^; β_3_ Gly^308^-Asn^421^; γ_2_ Ser^322^-Ala^408^; δ Asp^312^-Asp^401^. These constructs were cloned into the pHLsec vector (*52*) after its N-terminal signal sequence and without tags or where appropriate with a C-terminal 1D4 purification tag derived from bovine rhodopsin (TETSQVAPA) that is recognized by the Rho-1D4 monoclonal antibody (University of British Columbia) (*53*), as stated in relevant Materials and Methods sections.

### GABA_A_ receptor expression and purification for NB library

To generate an NB library, we recombinantly expressed and affinity-purified GABAA receptors containing either α_2_β_3_ or α_3_β_3_ subunits. The receptors were purified via a 1D4 tag present on the α subunit to ensure that both α and β subunits were included in each GABAA receptor pentamer because β_3_ subunits, but not α_2_ or α_3_ subunits, can form stable homopentamers (*54*). The long ∼100– to 150–amino acid M3-M4 ICDs were replaced with glvi sequences (SQPARAA) from the prokaryotic homolog *Gloeobacter violaceus* (*23*), and the TMDs were shielded by lauryl maltose neopentyl glycol detergent to ensure that NBs raised were focused against the large exposed ECDs of the receptors. GABA_A_R protein preparation was exactly as previously described for an equivalent α_1_β_3_ sample used to raise NBs in llamas (*55*).

### NB library: Selection, expression, and purification

Camelid NBs targeting the α_2_β_3_/a3β_3_-GABA_A_ receptors were generated using established protocols (*56*). One llama was immunized six times with the recombinant 1D4-tagged human α_2_β_3_ or a3β_3_ GABA_A_ receptor. After generating a library for each receptor, NBs were selected by phage display using two difference approaches: by passive adsorption of the GABA_A_ receptor pentamers or by chemical biotinylation followed by capture of the receptors on neutravidin-coated plates. During discovery, the β_3_-GABA_A_ receptor was added in solution to prevent the selection of β_3_-GABA_A_ receptor binding NBs. Selections were performed in 10 mM Hepes (pH 7.2), 150 mM NaCl, 0.007% decyl maltose neopentyl glycol (DMNG), and 0.00018% cholesterol hemisuccinate (CHS) either in the presence of GABA or gabazine. After selection, 48 clones from each selection round were screened. They were all screened in enzyme-linked immunosorbent assays using both solid phase-coated α_2_β_3_ or α_3_β_3_ versus β_3_ GABA_A_ receptors and neutravidin-captured biotinylated receptors. Positive clones were sequenced.

NBs were subcloned into the pHLsec vector (*52*) between the N-terminal signal sequence and a C-terminal His6 purification tag, between the N-terminal signal sequence plus an SBP tag (MDEKTTGWRGGHVVEGLAGELEQLRARLEHHPQGQREP) and a C-terminal His6 tag, or between the N-terminal signal sequence and a C-terminal mV-His8 (*33*) tag depending on downstream requirements. A total of 25 ml of Expi293F suspension cells (Thermo Fisher Scientific) grown to densities of 4 × 10^6^ cells/ml in Expi Expression Medium (Gibco), shaking at 130 rpm, 37°C, and 8% CO_2_ was transfected with 50 μg of DNA/200 μg of PEI Max. Cells were centrifuged and resuspended in 50 ml of fresh medium the following day, and supernatant was harvested 6 days later with viability typically at ∼70 to 80%. NBs were purified by addition of 5 ml of binding buffer [50 mM tris (pH 7.8) and 0.3 M NaCl] and 0.4 ml of nickel agarose resin, gently rotated at 4°C for 1 hour. After washing and imidazole elution [phosphate-buffered saline (PBS) containing 20 mM tris (pH 8.0), 0.5 M NaCl, and 0.25 M imidazole], NBs were concentrated and buffer exchanged by ultrafiltration using 10 -kDa cutoff membranes (Amicon) or size exclusion chromatography into PBS or external electrophysiology solution (see below for composition). Stocks were concentrated to 5 to 10 mg/ml, snap frozen in liquid nitrogen, and stored at −80°C.

### On-cell binding assays

Adherent HEK293T cells (American Type Culture Collection) were grown at 37°C and 5% CO_2_ in Dulbecco’s modified Eagle’s medium (DMEM; Sigma-Aldrich) supplemented with l-glutamine, nonessential amino acids, and 1% fluorescence correlation spectroscopy. After trypsinization, 50,000 cells were seeded per well of black 96-well microplates (Greiner) precoated with 0.01% (w/v) poly-l-lysine (PLL; Sigma-Aldrich). Eighteen hours postseeding, relevant cell wells were cotransfected with 30 μl of transfection cocktail containing 0.2 μg of plasmids encoding α_X_glvi plus β_3_glvi subunits at a 1:1 ratio and 0.4 μg of PEI (Polysciences).

Thirty hours posttransfection, plates were incubated on ice for 10 min, medium was removed by aspiration, and cells (whether in transfected or untransfected wells) were washed twice with 200 μl of PBS containing 0.5% (w/v) bovine serum albumin (PBS/BSA) and then incubated with the NB of interest. For single binding readouts, unpurified NB-mV in medium was used. For generating binding curves, purified SBP-tagged NBs were diluted in PBS/BSA. Untransfected cells were incubated with NB in medium or 20 μM purified NB for nonspecific binding (NSB) readouts. After aspiration and washing, well fluorescence was measured. For SBP-NBs, cells were further incubated with a 1/250 dilution of DyLight488-conjugated Streptavidin (Strep488, Thermo Fisher Scientific) in PBS/BSA before washing and relative fluorescence measured using a Tecan Infinite Pro plate reader (Tecan), with excitation/emission wavelength of 485/525 nm, integration time of 20 μs, and gain set to optimal. For initial library screening, refractory units (RFU) fold gains were calculated by dividing RFU of transfected cells by RFU of untransfected cells to obtain a readout of on-target NB binding. For binding curves, it was assumed that fluorescence intensity was proportional to on-cell surface GABA_A_ receptor binding, and data were fitted on GraphPad Prism (version 10.2.3) using nonlinear regression analysis with the following equation: *B* = (*B*_max_ × [NB])/([NB] × *K*_d_), where *B* is binding in RFU, *B*_max_ is maximal binding, and *K*_d_ is the NB dissociation constant. Inputting [NB] and *B* allowed calculation of *B*_max_ and *K*_d_. The former was used to normalize individual binding curves, enabling comparison of repeats. The latter quantified on-cell NB affinity for GABA_A_ receptors.

### GABA_A_ receptors for cryo-EM: Expression, purification, and reconstitution into nanodisks

Four protein samples were prepared: α_2_glvi-1D4 or α_3_glvi-1D4 plus β_3_bril transfected at 1:6 and α_2_glvi or α_3_glvi plus β_3_bril and γ_2_glvi-1D4 transfected at 1:1:1. Protein was prepared exactly as previously described (*34*). Final protein concentrations were ∼1 mg/ml for α_X_β_3_ preps and ∼0.5 mg/ml for α_X_β_3_γ_2_ preps.

### Cryo-EM grid preparation, data acquisition, and map processing

Elutions were used directly for cryo-EM grid preparation. For α_2_/α_3_β_3_γ_2_-GABA_A_ receptor grids, GABA was added at 200 μM. Megabody (Mb)F3 [previously described (*34*), for particle tumbling] was added at twofold molar excess, and NBs were added at 5 to 7 μM. For α_2_/α_3_β_3_-GABA_A_ receptor grids, histamine at 1 mM was also included, and MbF3 was replaced by Mb25 [previously described (*25*), for particle alignment] at fourfold molar excess. For grid preparation, 3 μl of sample was applied onto glow-discharged gold R1.2/1.3 300 mesh UltraAuFoil grids (Quantifoil) and then blotted for 3 s at blot forces typically ranging from −4 to +4 before plunge freezing the grids into liquid ethane cooled by liquid nitrogen. Plunge freezing was performed using a Vitrobot Mark IV (Thermo Fisher Scientific) at ∼100% humidity and 4°C. Cryo-EM data were collected either in the Department of Biochemistry, University of Cambridge or at the electron Bio-Imaging Centre (eBIC) at Diamond Light Source using the EPU software (version 3.7.0.6930). Collection parameters for all data sets are given in tables S3 and S4. Motion correction, contrast transfer function correction, and particle picking were performed using Warp1.0.9 (*57*) for α_2_/a3β_3_-GABA_A_ receptor collections or cryoSPARC (*58*) (version 4.4.1) for α_2_/α_3_β_3_γ_2_-GABA_A_ receptor collections. All subsequent particle processing was done in cryoSPARC. Local resolution maps were also generated in cryoSPARC based on the Fourier shell calculation (FSC) output calculated for voxels only within the mask output from the nonuniform refinement job used as the input for local resolution estimation. To generate maps colored by local resolution, the local resolution map along with the main map was opened in UCSF ChimeraX (*59*) (version 1.8) and processed using the surface color tool.

### Model building, refinement, validation, and presentation

Model building was conducted in Coot (*60*) (version 0.9.8.9) using the GABA/alprazolam-bound α_1_β_3_γ_2_-GABA_A_ receptor structure [Protein Data Bank (PDB) ID: 6HUO] as a template. The β_3_ and γ_2_ chains were adapted to match our construct design, while the α_1_ chain was point mutated to create α_2_/α_3_ chains. 6HUO was first fitted into the α_2_β_3_ + α_2NB_06 map using UCSF ChimeraX to build a working α_2_β_3_-GABA_A_ receptor model. This was subsequently used, in conjunction with 6HUO, to generate working α_3_β_3_, a2β_3_γ_2_, and a3β_3_γ_2_-GABA_A_ receptor models. Small-molecule ligands and their geometry constraint files were generated using the Grade Web Server (Global Phasing). Map resolutions were sufficient to allow ab initio building of the SQPARAA M3-M4 linkers for α_2_ and α_3_ chains, but not always for the less-well resolved γ_2_ chains. The larger and more disordered SQPAGT-BRIL-TGRAA M3-M4 linkers for the β_3_ chains were not modeled. Initial NB and Mb25 models were generated using AlphaFold2 (*61*) and then refined within respective maps using Coot. Model refinement was performed through iterative rounds of local/manual inspection and global real-space refinement, using Coot and Phenix (*62*) (version 1.21.1), respectively. Model geometry was evaluated using the MolProbity Web Server and the built-in Phenix validation tool (*62*, *63*). The results are summarized in tables S3 and S4. Measurements of pore diameters were generated using the HOLE plug-in in Coot (*64*). The following were generated or calculated using UCSF ChimeraX: structural overlays, using the Matchmaker function; Cα RMSD values, using the rmsd function; rotation angles, distance measurements, and structural presentations.

### Manual HEK cell preparation and electrophysiology

HEK293 cells were maintained at 37°C in DMEM supplemented with 10% (v/v) fetal bovine serum (FBS), penicillin-G (100 U/ml), and streptomycin (100 μg/ml; 95% air/5% CO_2_). During cell passage, 0.05% (w/v) trypsin-EDTA was used to dislodge cells. For the expression of recombinant GABA_A_Rs, murine subunit DNA clones (α_1–6_, β_2–3_, γ_2_, and δ; pRK5 vector) were used in transient transfections. Before transfections, HEK cells were seeded on PLL-coated glass coverslips. Cells were transfected with α_X_:β_X_:GFP, α_X_:β_X_:γ_2_:GFP, and α_X_:β_X_:δ:GFP cDNAs in ratios of 1:1:1 or 1:1:3:1, using a standard calcium-phosphate precipitation protocol with ∼6 μg of cDNA, 20 μl of 340 mM CaCl_2_, and 24 μl of 50 mM Hepes, 280 mM NaCl, and 2.8 mM Na_2_HPO_4_ per transfect. Electrophysiology was performed 16 to 30 hours after transfection.

Green fluorescent protein (GFP)–transfected HEK cells were identified with a Nikon ECLIPSE FN1 microscope before patching. Cells were perfused with Krebs solution containing 140 mM NaCl, 4.7 mM KCl, 1.2 mM MgCl_2_, 2.52 mM CaCl_2_, 11 mM glucose, and 5 mM Hepes (pH 7.4), and thin-walled patch pipettes (3 to 4 megohms) were filled with intracellular solution containing 120 mM KCl, 1 mM MgCl_2_, 11 mM EGTA, 30 mM KOH, 10 mM Hepes, 1 mM CaCl_2_, and 2 mM adenosine 5′-triphosphate (ATP)–K_2_ (pH 7.2). Cells were voltage clamped at −40 mV using an Axopatch 200B amplifier (Molecular Devices, USA). Whole-cell GABA_A_ currents were filtered at 5 kHz (−36 dB per octave), digitized at 50 kHz via a Digidata 1550B (Molecular Devices), and recorded to a PC using Clampex 10.7 (Molecular Devices). Series resistance was compensated at 70%, and only data with less than 20% change in series resistance during experiments were analyzed. NBs and all other compounds were made in Krebs solution and applied to cells using a fast Y-tube delivery system.

Peak currents were measured using ClampFit (Molecular Devices, v10.7), and Prism (GraphPad, v10.4) was used for statistical analysis, data fitting, and graphing. To generate inhibition concentration-response curves, data values were fitted to the following equation: *I*/*I*_max_ = 1 – [[*B*]*^n^*/(IC_50_*^n^* + [*B*]*^n^*)]. *I* represents the current response; [*B*] is the concentration of the NB; *n* is the slope coefficient; and IC_50_ is the concentration producing 50% inhibition of GABA current (inhibitory potency). Drug potentiation concentration response curves were generated by fitting data points using the Hill equation: *I*/ *I*_max_ = [1/(1 + (EC_50_/[A])*^n^*]. EC_50_ represents the concentration of the drug ([A]) inducing 50% of the maximal current evoked by a saturating concentration of the agonist, and *n* is the Hill coefficient. Potency values are presented as pIC_50_ or pEC_50_ ± SEM, and the mean value was transformed into a molar concentration according to the following: pIC_50_ = –logIC_50_, or pEC_50_ = –logEC_50_.

### Acute brain slice recording electrophysiology

All procedures involving animals complied with the ARRIVE guidelines [ARRIVE guidelines; National Centre for the Replacement, Refinement and Reduction of Animals in Research (NC3Rs)] and were performed in accordance with UK Home Office regulations under the Animals (Scientific Procedures) Act 1986, following ethical review by University College London (UCL)’s Animal Welfare and Ethical Review Bodies (PP6960143)*.* Mice (C57Bl/6J) were maintained under a standard 12:12-hour light/dark cycle and were allowed ad libitum access to food and water.

For acute brain slice preparation (*65*), adult male mice (2 to 4 months old) were anaesthetized with isoflurane and decapitated before rapid removal of the brain. Coronal sections (250 μm) containing either the hippocampus or amygdala were prepared using a Leica VT 1200 S vibroslicer with the brain immersed in ice-cold slicing solution composed of 130 mM k-gluconate, 15 mM KCl, 0.05 mM EGTA, 20 mM Hepes, 4 mM Na-pyruvate, 25 mM glucose, and 2 mM kynurenic acid (pH 7.4). Brain slices were subsequently transferred to a holding chamber incubated at 37°C where the slicing solution was slowly exchanged (over ∼1 hour) for artificial cerebrospinal fluid (aCSF) containing 125 mM NaCl, 2.5 mM KCl, 1.25 mM NaH_2_PO_4_, 26 mM NaHCO_3_, 2 mM CaCl_2_, 1 mM MgCl_2_, 25 mM glucose, and 2 mM kynurenic acid (pH 7.4 when bubbled with 95% O_2_ and 5% CO_2_). Slices were maintained in the holding chamber at room temperature (RT) until required for electrophysiology.

Recording from neurons in acute brain slices: Brain slices were superfused with RT aCSF supplemented with 2 mM kynurenic acid (Sigma-Aldrich) to block excitatory glutamatergic transmission and whole-cell recordings made from hippocampal DGGCs or BLA principal neurons visualized using infrared differential interference contrast optics. Patch pipettes had resistances of 2.5 to 4 megohms and were filled with an internal solution containing 140 mM CsCl, 2 mM NaCl, 10 mM Hepes, 5 mM EGTA, 2 mM MgCl_2_, 0.5 mM CaCl_2_, 2 mM NaATP, and 0.5 mM NaGTP (pH 7.3, osmolarity 300 ± 10 megohms). Membrane currents were recorded from neurons voltage clamped at −60 mV using a Multiclamp 700B amplifier (Molecular Devices). Currents were filtered at 2 kHz, digitized at 20 kHz via a Power1401 interface (Cambridge Electronic Design) and acquired using the WinEDR software (version 3.5.2, John Dempster, Strathclyde University). Series resistance was monitored throughout all recordings by measuring the membrane current responses to 10-mV hyperpolarizing voltage steps. If the series resistance varied by more than 30%, then cells were discarded. All solutions were bath applied.

For the analysis of GABA-mediated IPSCs, event detection was performed using the WinEDR software (version 3.5.2) using an amplitude-threshold crossing method. All detected events were manually checked before further analysis was carried out in WinWCP (version 4.8.6, John Dempster) to calculate event amplitudes and the frequency of IPSCs under different recording conditions. All validated IPSCs were included for the analyses of event amplitude and frequency, while only “clean” events that showed monotonic rises and uncontaminated decay phases were used for kinetic analysis (>50 events under each condition). These were aligned on their rising phases and averaged to produce mean IPSC waveforms. Synaptic current decay times were characterized using the weighted decay tau (τ_w_) calculated by fitting a biexponential curve to the decay phase of the mean IPSC waveform and applying the equation

$$\tau_{w}=\frac{A_{1}.\tau_{1}+A_{2}.\tau_{2}}{A_{1}+A_{2}}$$

where τ_1_ and τ_2_ represent time constants for the two exponential components of the decay phase and *A*_1_ and *A*_2_ are their relative amplitude contributions.

### Primary mouse cortical cultures

Experimental procedures and animal use were performed in accordance with UK Home Office regulations of the UK Animals (Scientific Procedures) Act 1986 and Amendment Regulations 2012, following ethical review by the University of Cambridge Animal Welfare and Ethical Review Body. All animal procedures were authorized under personal and project licenses held by O.P. and A.S.

Mice were housed on a 12-hour light/dark cycle at 19° to 23°C and were provided with food and water ad libitum. Experiments were carried out on offsprings of wild-type C57BL/6J mice (Harlan, Bicester, UK or Central Animal Facility, Physiological Laboratory, Cambridge University). Pups were euthanized by decapitation at postnatal day 0 (P0) to P1 under Home Office project license PP0499088. MEA data include mice cultured from three separate wild-type litters (*n* = 3).

Before dissection, MEA chips are first cleaned using water and 3 to 5% Tergazyme (Alconox) to break down debris residue. Clean MEAs are then sterilized in the autoclave on an instruments cycle (120°C for at least 30 min). To treat the MEA chips for cell plating, 5 μl of heavyweight PLL (Sigma-Aldrich) is slowly dropped on the surface of the grid and left to incubate at 37°C with 5% CO_2_/95% air for 30 min. After incubation, 15 μl of Dulbecco’s Phosphate-Buffered Saline (DPBS; Gibco) is used to wash away the PLL two times and left to dry in the hood. When completely dry, 7 μl of laminin (Sigma-Aldrich) is slowly dropped onto the center of the grid and incubated for at least 1 hour before cell plating.

For the murine primary cortical cultures, postnatal (P0 or P1) mice were euthanized by decapitation under the Home Office project license PP0499088. The dissected cortices were initially dissociated chemically with papain at 37°C for 20 min. To stop this reaction, Neurobasal-A medium (Gibco) supplemented with B27 (Gibco), 125 μl of GlutaMAX (Gibco), and 4% FBS (Gibco) was added, followed by manual dissociation of the cells and centrifugation at 4 relative centrifugal force (RCF) for 7 min. Deoxyribonuclease (50 mg/ml; Roche) was added for 5 min to prevent cell clumping before the supernatant was removed, and the cells were resuspended. The cellular yield was estimated, and 80,000 to 100,000 cells were plated onto the pretreated MEA grids. The cultures were covered with zero-evaporation lids (*66*) and incubated at 37°C with 5% CO_2_/95% air for 30 min. Subsequently, 600 μl of the NBA/B27/GlutaMAX mixture was added to the MEA. The medium was partially exchanged three times per week, with one-third of the volume exchanged each time.

### Immunofluorescence staining of cortical cultures

A total of 120,000 to 150,000 cells was plated on 15-mm-diameter coverslips for immunostaining using the Beaudoin *et al.* protocol (*67*). Cells were plated in 400 μl of NBA/B27 10% FBS medium. At DIV1, 600 μl of NBA/B27/GlutaMAX medium was added. One-third of the medium was exchanged every other day for fresh NBA/B27/GlutaMAX medium to maintain the cells. For staining, coverslips were washed with 1 ml of 1× PBS warmed at 37°C and then incubated with 500 μl of 4% paraformaldehyde in 1× PBS, followed by three washes with 1 ml of 1× PBS and quenching with 1 ml of 0.1 M glycine in 1× PBS for 10 min. Cells were permeabilized with 0.5% Triton in 1× PBS for 10 min, followed by blocking with 0.5% BSA in 1× PBS for 30 min at RT. The primary antibodies rabbit anti-alpha2 (Synaptic Systems) and chicken anti-MAP2 (Abcam) were diluted at 1:250 in 0.5% BSA in 1× PBS, and cells were incubated for 30 min at RT. Primary antibodies were washed twice with 1 ml of 0.5% BSA in 1× PBS during 5 min, and secondary antibodies donkey anti-rabbit AF488 (Thermo Fisher Scientific) and goat anti-chicken Alexa Fluor 405 (Abcam) were diluted at 1:500 in 0.5% BSA in 1× PBS added for 30 min at RT. The coverslips were washed twice with 1 ml of 0.5% BSA in 1× PBS during 5 min and mounted on glass slides using Fluoroshield (Sigma-Aldrich) and left to dry for a few hours before observation under the microscope.

### Microelectrode array recording (MEA) and analysis

The MEA2100 system and MCRack software (version 4.6.2, Multi Channel Systems) were used to record spontaneous activity at 25 kHz. MEA chips with 60 extracellular electrodes were used including one internal reference electrode (12 μm diameter; 200 μm spacing; 2D, 60MEA200/30iR-ITO-gr). Each recording had a duration of 10 min. Before each recording, electrodes that had noise levels exceeding 50 μV were grounded. Activity of the MEAs was monitored without recording for a period of 90 to 120 s before recording to allow electrodes to conFig. to the recording pins. Each culture was recorded at baseline before treatments. After an incubation period and NB or control treatments, cultures were immediately recorded for posttreatment. Follow-up recordings were then taken after 2 and 24 hours following the treatment. NBs were diluted with 100 μl of fresh NB-B27, then filtered, and further diluted with existing medium in the MEA well (500 μl) to give a final concentration of 5 μM.

The spike rate (spikes per s) was calculated for each electrode before taking the mean spike rate across electrodes. Population bursts were detected semiautomatically based on the interspike interval (ISI) distribution using the ISI*_N_* method (*68*). The *N* parameter, which determines the minimum number of spikes required to infer a burst, was set to 10 spikes. The minimum number of channels required to infer a population burst was set to three. The ISI between every 10th spike across all electrodes was calculated. Bursts are inferred where the ISI*_N_* falls shorter than a threshold determined by identifying the lowest point between two peaks in the ISI*_N_* distribution that corresponds to high-frequency spiking activity.

The spike time tiling coefficient (*69*) was used to quantify the correlation in spike timing between all pairs of electrodes. A synchronicity of 10 ms was used, which corresponds to the temporal lag with which spike times are considered overlapping and thus correlated. For each electrode, we computed the mean of all its significant correlation weights, referred to as node strength. Significance of connections was inferred via permutation of spike trains to create a null distribution for each interelectrode pairwise correlation and an alpha level of 5% was used. Statistical analysis and corresponding visualizations in this project were performed in GraphPad Prism (version 10.4.1), MATLAB (version 24.2: R2024b) and R software (version 4.3.1).

### BLA immunohistochemistry

The experiment was conducted in accordance with the UK 1986 Animals (Scientific Procedures) Act following ethical review by the University of Cambridge Animal Welfare and Ethical Review Body. Mice were anaesthetized using isoflurane and cardiac perfused with PBS, followed by PBS + 4% paraformaldehyde. Fixed brains were excised, stored in PBS + 4% paraformaldehyde for a further 24 hours, and then transferred into 30% sucrose for 5 days. Brains were sectioned by cryostat, and 80-μm-thickness sagittal sections of the BLA were adhered to microscope slides for staining. Slices were incubated in primary antibody at room temperature for 1 hour, followed by PBS washes, incubation with secondary antibody for 1 hour, further PBS washes, and then application of ProLong Gold Antifade Mountant (Invitrogen, P10144) and coverslip. Primary antibodies: GABA_A_-R α_1_ (1:500; Millipore, 06–868, rabbit), GABA_A_-R α_2_ (1:250; Synaptic Systems, 224103, rabbit), α_2NB_00 fused via C to N terminus of human immunoglobulin G (IgG) [100 nM (20 μg/ml), made in-house], and VGAT (1:250; SYSY Antibodies, 131011, mouse). Secondary antibodies: Anti-rabbit IgG AF488 (1:500; Abcam, ab150077, goat); Anti-rabbit IgG Alexa Fluor 647 (1:500; Invitrogen, A21245, goat); Anti-human IgG AF488 (1:500; Invitrogen, A11013, goat); and Anti-mouse IgG Alexa Fluor 568 (1:500; Invitrogen, A11004, goat).

### EPM rat experiments

The experiment was conducted in accordance with the UK 1986 Animals (Scientific Procedures) Act following ethical review by the University of Cambridge Animal Welfare and Ethical Review Body under the project license number PP0463796. Two separate cohorts of 40 male Sprague-Dawley rats (*N* = 80) from Charles River Laboratories (UK) were tested sequentially to ensure replicability.

Stereotaxic surgeries were conducted under isoflurane anesthesia (O_2_: 2 liters/min, 5% isoflurane for induction, and 2 to 3% for maintenance) as previously described (*70*). Rats were positioned on the stereotaxic frame (David Kopf Instruments, Tujunga, CA, USA) and were implanted bilaterally with 22-gauge cannulae (Plastics One, Roanoke, VA, USA) positioned to lie 2 mm above the BLA (from bregma: AP –2.6; ML ±4.9; from skull: DV –6.8, with the incisor bar set at −3.3 mm) (*71*). Cannulae were held in place using dental acrylic anchored to stainless steel screws tapped into the frontal and parietal bones of the skull. Obturators (Plastics One) were placed in the cannulae to maintain patency. Rats were treated with an analgesic agent (Metacam; 1 mg/kg) administered subcutaneously immediately before surgery and orally (1 mg/kg daily) for the five consecutive days. After at least a week, each rat underwent two habituation infusion sessions, during which the obturators were removed, sterile injectors with tips protruding by 2 mm were inserted, and infusion pumps were run for 100 s. Starting the next day, rats were tested on the EPM following an intracranial infusion (0.5 μl per side at 0.3 μl/min, 1-min diffusion period) of one of the test compounds (0.9% saline buffer vehicle, diazepam (14 mM; in 40% propylene glycol/10% EtOH in 0.9% saline), bicuculline (2.7 mM in 0.0002% glacial acetic acid in 0.9% saline), α_2NB_04 (250 μM), α_2NB_04 (low: 40 μM; high: 250 μM), α_3NB_83 (250 μM), or α_2NB_25 (40 μM). Treatments were randomized across subjects. After the infusion, rats were immediately habituated to the EPM room for 5 min before the test. For the EPM test, animals were placed in the central platform, facing the same open arm, and allowed to freely explore for 5 min while their behavior was recorded from an overhanging camera (*72*, *73*). Time spent, distance traveled, and entries made into each of the three classes of compartments [open arm (OA), closed arm (CA), and central platform] were automatically recorded by the tracking software. The percentage of time spent in the OA was the main behavioral index of anxiety [%OA = (time OA × 100%)/(time OA + time CA) × 100%], whereby a greater %OA indicated less anxiety (*32*, *72*–*74*). Ninety min after the EPM, rats were anesthetized with pentobarbital and perfused. Brains were fixed, cryoprotected, and sliced at 35 μm for immunohistochemistry. BLA sections were stained with cresyl violet and imaged under brightfield to confirm cannula placement.

The EPM is elevated 50 cm above the floor and comprises two open arms (45 × 10 cm), two closed arms (45 × 10 × 45 cm), and a central platform (10 × 10 cm). The luminosity was measured in each portion of the EPM, and the light was adjusted to reach an intensity of 40 lux in the central platform, 50 lux in the open arms, and 30 lux in the closed arms (*73*). The tests were conducted as previously described (*73*, *75*) during the dark phase of the dark/light cycle between 8 and 11 a.m.

### Quantification and statistical analysis

For the on-cell binding assay: Histograms and graphs are presented as mean or mean ± SEM, and individual points are shown where possible (*n* = 3). Statistical analysis was not performed. Each *n* represents a separate experiment.

For HEK293T whole-cell patch-clamp recordings: Histograms and graphs are represented as mean values ± SEM, and individual points are shown where possible (*n* = 3 to 6 for Fig. 2A; *n* = 3 for Fig. 3A; *n* = 5 for Fig. 4A; *n* = 3 for Fig. 4, C and D; *n* = 3 for Fig. 5, A, C, and D; *n* = 4 for Fig. 7A). Datasets were compared using Student’s *t* tests or analyses of variance (ANOVAs) with Tukey’s multiple-comparison post hoc tests where appropriate. Statistical labels on graphs are defined as n.s., not significant, *P < 0.05, **P < 0.01, and ****P < 0.0001. Each *n* represents a separate cell recording.

For acute brain slice electrophysiology: Histograms and graphs are represented as mean values ± SEM, and individual points are shown where possible (*n* = 8 for Fig. 3F, *n* = 5 for Fig. 3, G and H; *n* = 6 for Fig. 4, G, H, J, and K; *n* = 5 for Fig. 5, G, H, J, and K; *n* = 6 for Fig. 7, B, D, and E). The Shapiro-Wilk test was used to check for normality with subsequent pairwise comparisons made using either a paired two-tailed *t* test or a Wilcoxon matched-pairs signed-rank test as appropriate. All statistical analyses were carried out using GraphPad Prism (version 10.4.1, GraphPad Software), with the threshold for statistical significance set at *P* < 0.05. Each *n* represents a separate cell recording.

For the cortical culture MEA recordings: The normality of data was assessed with the Shapiro-Wilk test and by visually inspecting Q-Q plots. The normality of residuals and sphericity was further evaluated using Mauchly’s test. Because these assumptions were violated, a nonparametric approach was adopted, consistent with previous brain network research (*76*). To assess statistical significance, the Kruskal-Wallis test, a nonparametric one-way ANOVA test calculated on ranks rather than raw data, was applied. The Dunn’s post hoc multiple-comparison test was used to control for familywise error rate, and corrected individual values were further examined between comparisons. Each *n* represents a separate neuronal culture.

For the BLA immunohistochemistry: Histogram is represented as mean values ± SEM, and individual points are shown (*n* = 3 for α_2_ and *n* = 4 for α_1_ in Fig. 7D). Statistical comparison used unpaired, two-tailed *t* test. Threshold for statistical significance set at *P* < 0.05. For all staining experiments, *n* = 3 to 4 repeats were performed with each *n* representing a separate animal and brain slice used in an independent staining experiment.

For the EPM: Values presented as mean ± SEM (*n* = 4 to 12 rats). All behavioral variables were analyzed using a univariate ANOVA with treatment as a fixed between-subject factor. Significant main effects of treatment were followed by Sidak-corrected group comparisons. Planned comparisons were performed between controls (vehicle and silent NB groups pooled) and all treatment groups. Normality was assessed with the Shapiro-Wilk test, and if violated, analyses were conducted on log transformed data. Each *n* represents a separate rat.
