# Supplementary material for: Determining the molecular and physiological actions of subtype-selective nanobodies of GABAA receptors
Source: Sci Adv. 2026 Jul 29;12(31):eaeg3548. doi: 10.1126/sciadv.aeg3548 (PMC13418544; doi:10.1126/sciadv.aeg3548)
Supplement: Supplementary file 1 — Figs. S1 to S7 Tables S1 to S5 [file sciadv.aeg3548_sm.pdf]

Science Advances
AAAS

Supplementary Materials for

Determining the molecular and physiological actions of subtype-selective nanobodies of $GABA_A$ receptors

Jose Enrique Gonzalez-Prada *et al.*

Corresponding author: Trevor G. Smart, t.smart@ucl.ac.uk; Paul S. Miller, pm676@cam.ac.uk

Sci. Adv. **12**, eaeg3548 (2026)
DOI: 10.1126/sciadv.aeg3548

This PDF file includes:

Figs. S1 to S7
Tables S1 to S5

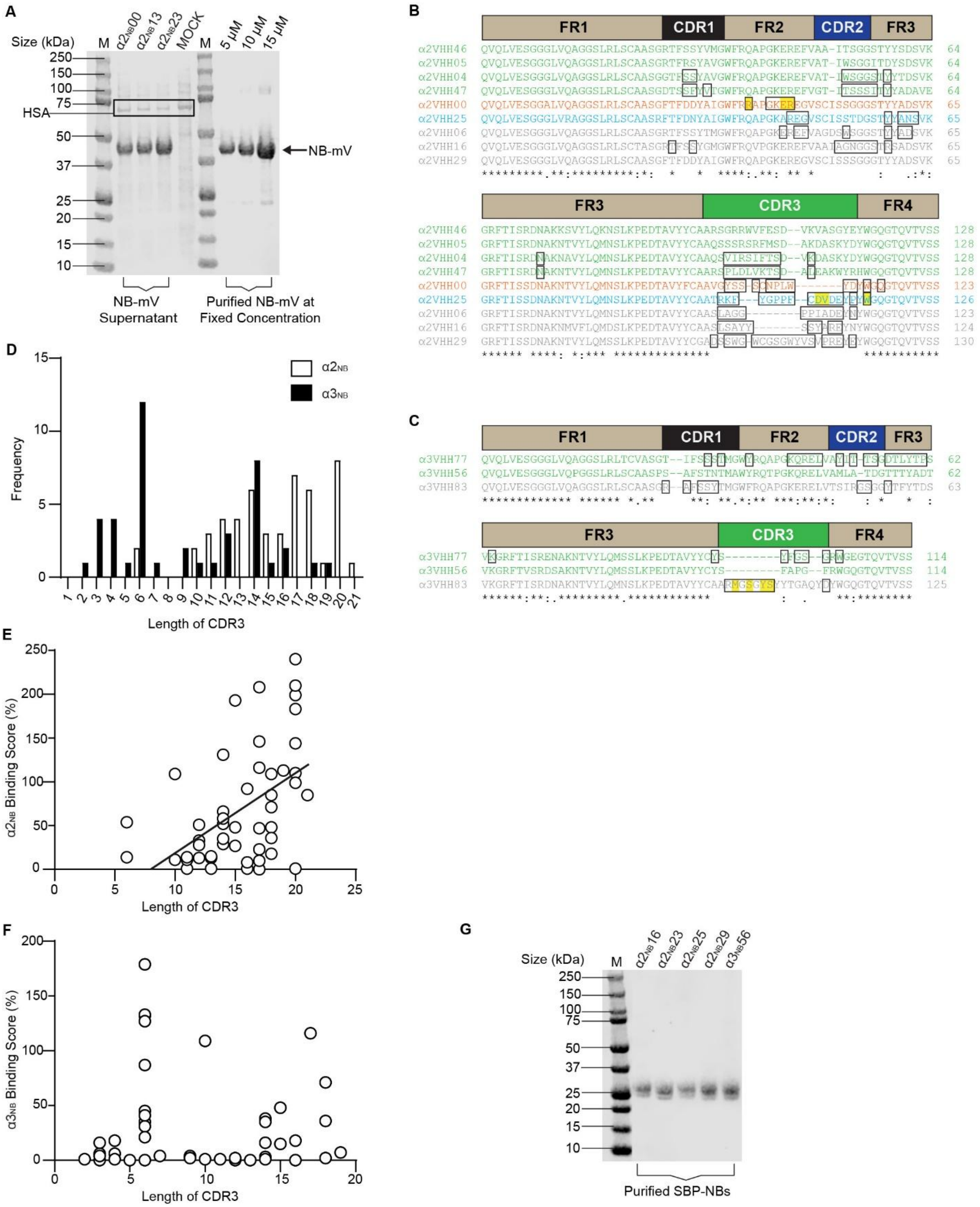
A
Size (kDa)
M
MOCK
5 μM
10 μM
15 μM
HSA
NB-mV
NB-mV Supernatant
Purified NB-mV at Fixed Concentration
B
FR1
CDR1
FR2
CDR2
FR3
CDR3
FR4
C
D
Frequency
Length of CDR3
E
Length of CDR3
F
Length of CDR3
G
Size (kDa)
Purified SBP-NBs

Fig. S1. NB protein sequence, expression and binding properties. (**A**) Representative expression media for three NBs run on Coomassie-stained SDS-PAGE gels. All 10 µl loads. (**B**) ClustalW protein sequence alignment for key characterized NBs from this study. For NBs with cryo-EM structures, all residues within 4 Å of the $GABA_A$ receptor interface are highlighted by black boxes. Residues referred to in the main text are boxed and highlighted in orange. (**C**) ClustalW protein sequence alignment for key characterized NBs from this study. For NBs with cryo-EM structures, all residues within 4 Å of the $GABA_A$ receptor interface are highlighted by black boxes. Residues referred to in the main text are boxed and highlighted in orange. (**D**) Histogram comparing the frequencies of each CDR3 length for the α2 and α3 NBs. (**E**) Binding score versus CDR3 length plot for the α2 NBs, fitted with Spearman's rank correlation line, r_s = 0.49, n = 50, P < 0.001. (**F**) Binding score versus CDR3 length plot for the α3 NBs. Spearman's rank correlation line not shown, r_s = 0.04, n = 43, P = 0.78. (**G**) Representative purified SBP N-terminal fused NBs run on Coomassie stained SDS-PAGE gels after nickel bead purification. All 0.5 µg loads.

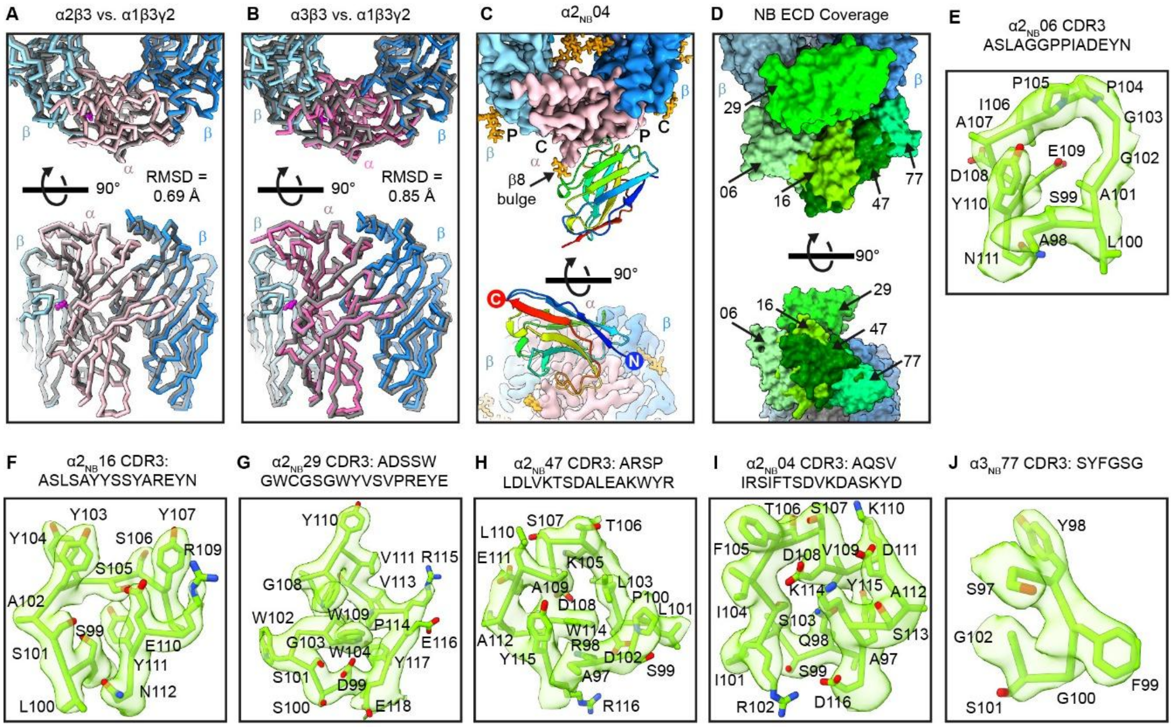

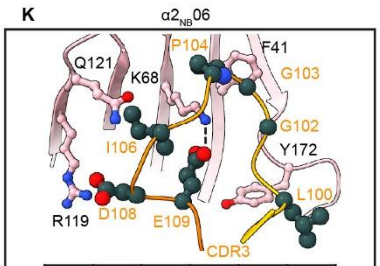

α1	α2	α3	α4	α5	α6
K42	F41	K66	K40	R45	K40
S69	K68	T93	T67	S72	T67
T122	Q121	V146	M120	E125	M120
R173	Y172	L197	K171	N176	K171

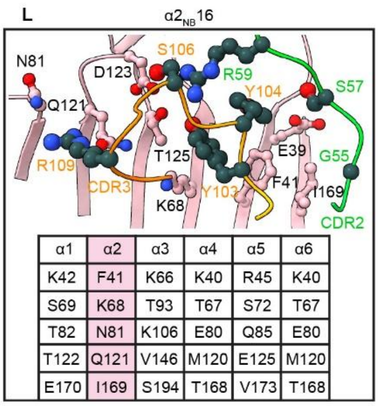

α1	α2	α3	α4	α5	α6
K42	F41	K66	K40	R45	K40
S69	K68	T93	T67	S72	T67
T82	N81	K106	E80	Q85	E80
T122	Q121	V146	M120	E125	M120
E170	I169	S194	T168	V173	T168

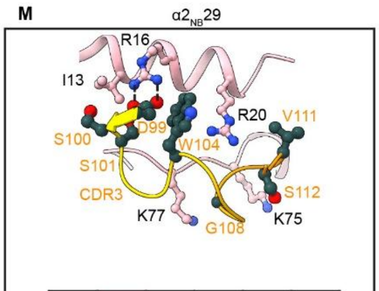

α1	α2	α3	α4	α5	α6
V14	I13		N12		N12
	R20		S19	G24	N19
	K77	D102	D76		G76

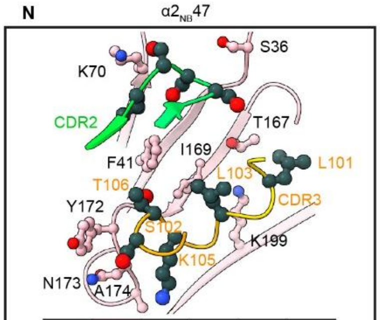

α1	α2	α3	α4	α5	α6
R37	S36	A61	P35	R40	A35
K42	F41	K66	K40	R45	K40
V168	T167	V192	I166	V171	I166
E170	I169	S194	T168	V173	T168
R173	Y172	L197	K171	N176	K171
E174	N173	G198	G172	G168	G172
P175	A174	K199	P173	S178	P173
S200	K199	T224	S198	T203	S198

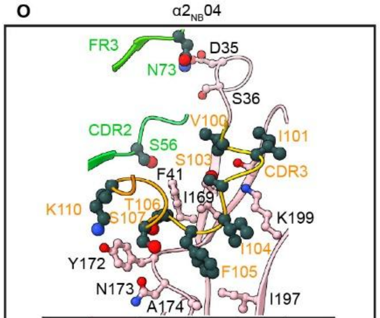

α1	α2	α3	α4	α5	α6
R37	S36	A61	P35	R40	A35
K42	F41	K66	K40	R45	K40
V168	T167	V192	I166	V171	I166
E170	I169	S194	T168	V173	T168
R173	Y172	L197	K171	N176	K171
E174	N173	G198	G172	G168	G172
P175	A174	K199	P173	S178	P173
V198	I197	V222	V196	C201	V196
S200	K199	T224	S198	T203	S198

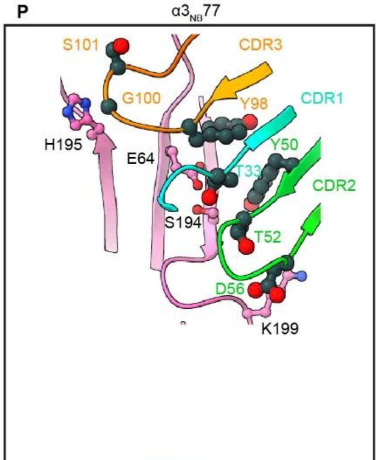

α1	α2	α3	α4	α5	α6
K71	K70	H95	I69	K74	T69
E170	I169	S194	T168	V173	T168
P175	A174	K199	P173	S178	P173

Fig. S2. Silent and "near" silent NB binding modes. (**A-B**) Top-down and side view C_α backbone representations of the β-α-β ECD region from (**A**) α2β3 or (**B**) α3β3, overlaid with a structural alignment of the equivalent region from the GABA-bound α1β3γ2 receptor (PDB: 6HUO – grey), showing the same arrangement. GABA bound to the α2β3 model shown in magenta. (**C**) Top-down and side views of the α2β3 cryo-EM map bound by $\alpha2_{NB}04$ (ribbon representation, rainbow colouring, blue N-terminus to red C-terminus). Subunit ECD Principal (P) and Complementary (C) faces are indicated. (**D**) Top-down and side views of the surface representation of an αXβ3 cryo-EM map superposed with five NBs that cover the five binding locations/modes in different shades of green, showing that the complete upper half of the α-subunit ECD is masked. (**E-J**) CDR3 electron density, protein sequence and model build for (**E**) $\alpha2_{NB}06$, (**F**) $\alpha2_{NB}16$, (**G**) $\alpha2_{NB}29$, (**H**) $\alpha2_{NB}47$, (**I**) $\alpha2_{NB}04$, (**J**) $\alpha3_{NB}77$. (**K-P**) NB binding modes and tabulated lists of α-subunit amino acid variants for α-subunit residues within 4Å of the bound NB – only amino acid positions that vary between α-subunit subtypes are tabulated: (**K**) $\alpha2_{NB}06$, (**L**) $\alpha2_{NB}16$, (**M**) $\alpha2_{NB}29$, (**N**) $\alpha2_{NB}47$, (**O**) $\alpha2_{NB}04$, (**P**) $\alpha3_{NB}77$. Nitrogen atoms – blue, oxygen atoms – red.

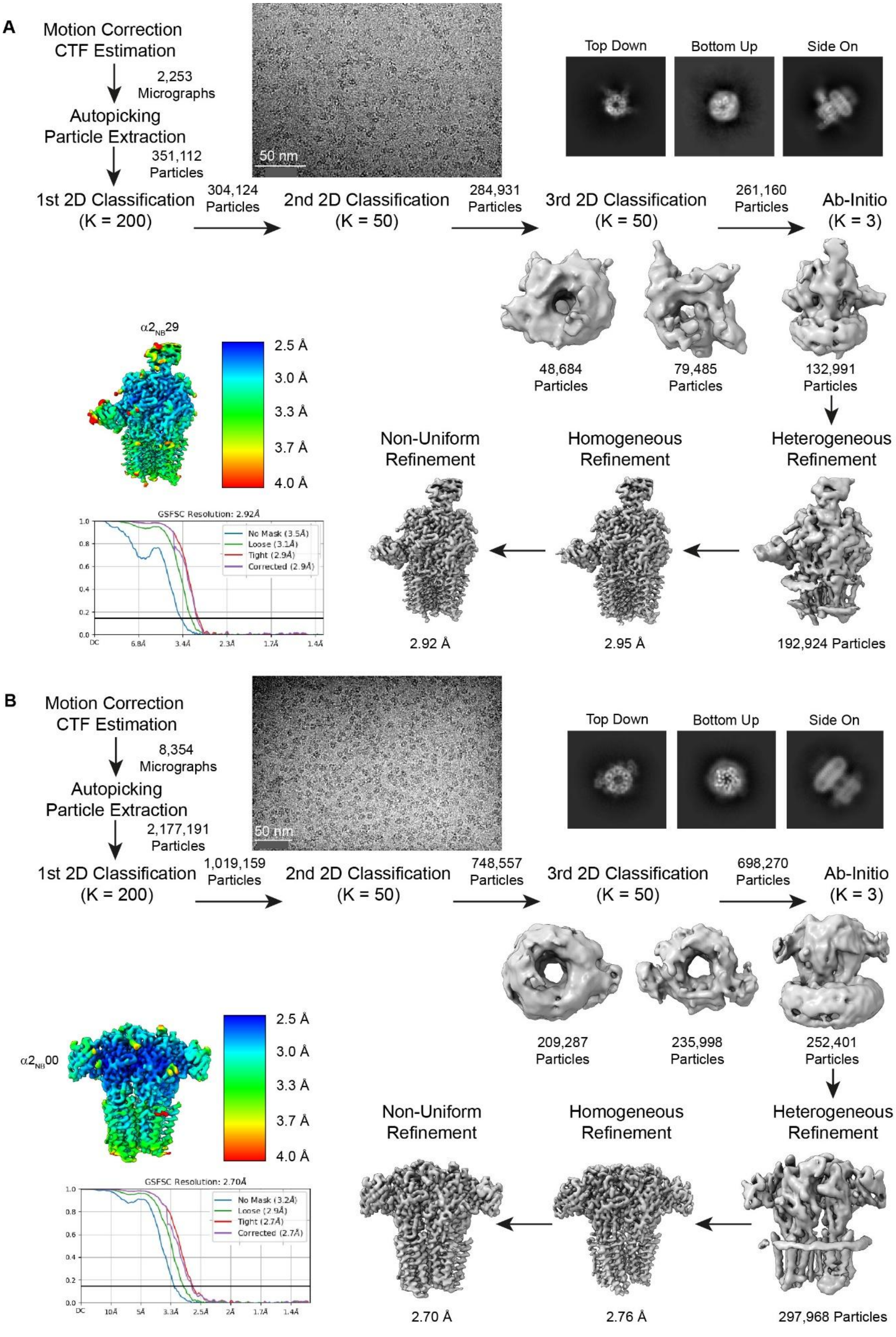
A
Motion Correction
CTF Estimation
2,253
Micrographs
Autopicking
Particle Extraction
351,112
Particles
50 nm
Top Down
Bottom Up
Side On
1st 2D Classification
(K = 200)
304,124
Particles
2nd 2D Classification
(K = 50)
284,931
Particles
3rd 2D Classification
(K = 50)
261,160
Particles
Ab-Initio
(K = 3)
48,684
Particles
79,485
Particles
132,991
Particles
α2NB29
2.5 Å
3.0 Å
3.3 Å
3.7 Å
4.0 Å
Non-Uniform
Refinement
Homogeneous
Refinement
Heterogeneous
Refinement
GSFSC Resolution: 2.92Å
No Mask (3.5Å)
Loose (3.1Å)
Tight (2.9Å)
Corrected (2.9Å)
2.92 Å
2.95 Å
192,924 Particles
B
Motion Correction
CTF Estimation
8,354
Micrographs
Autopicking
Particle Extraction
2,177,191
Particles
50 nm
Top Down
Bottom Up
Side On
1st 2D Classification
(K = 200)
1,019,159
Particles
2nd 2D Classification
(K = 50)
748,557
Particles
3rd 2D Classification
(K = 50)
698,270
Particles
Ab-Initio
(K = 3)
209,287
Particles
235,998
Particles
252,401
Particles
α2NB00
2.5 Å
3.0 Å
3.3 Å
3.7 Å
4.0 Å
Non-Uniform
Refinement
Homogeneous
Refinement
Heterogeneous
Refinement
GSFSC Resolution: 2.70Å
No Mask (3.2Å)
Loose (2.9Å)
Tight (2.7Å)
Corrected (2.7Å)
2.70 Å
2.76 Å
297,968 Particles

Fig. S3. Cryo-EM processing workflow for an αβ and αβγ receptor. (**A**) Image processing workflow for α2β3 bound by $\alpha 2_{NB}29$ in cryoSPARC from micrographs through 2D classes and 3D ab initio model to final model after refinement rounds with global and local map resolution shown, applicable to any αβ receptor. (**B**) Image processing workflow for α2β3γ2 bound by $\alpha 2_{NB}00$ in cryoSPARC from micrographs through 2D classes and 3D ab initio model to final model after refinement rounds with global and local map resolution shown, applicable to any αβγ receptor.

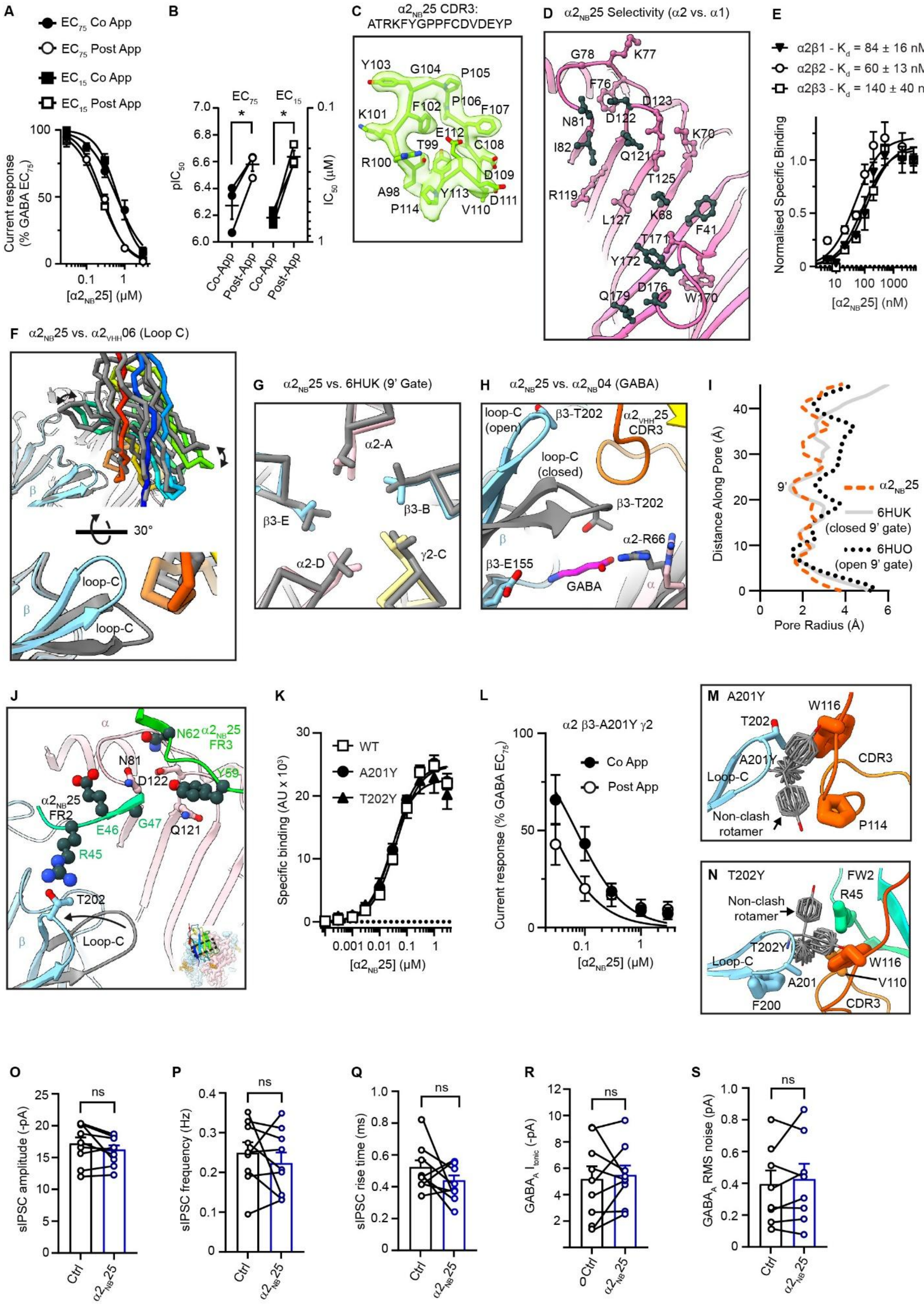
A
EC75 Co App
EC75 Post App
EC15 Co App
EC15 Post App
Current response (% GABA EC75)
[α2NB25] (μM)
B
EC75
EC15
pIC50
IC50 (μM)
Co-App
Post-App
C
α2NB25 CDR3:
ATRKFYGPPFCDVDEYP
Y103
G104
P105
P106
F107
K101
F102
E112
T99
C108
R100
D109
A98
Y113
D111
P114
V110
D
α2NB25 Selectivity (α2 vs. α1)
G78
K77
F76
D123
N81
D122
K70
I82
Q121
T125
R119
K68
L127
F41
T171
Y172
D176
Q179
W170
E
α2β1 - Kd = 84 ± 16 nM
α2β2 - Kd = 60 ± 13 nM
α2β3 - Kd = 140 ± 40 nM
Normalised Specific Binding
[α2NB25] (nM)
F
α2NB25 vs. α2VHH06 (Loop C)
β
30°
loop-C
G
α2NB25 vs. 6HUK (9' Gate)
α2-A
β3-E
β3-B
α2-D
γ2-C
H
α2NB25 vs. α2NB04 (GABA)
loop-C (open)
β3-T202
α2VHH25 CDR3
loop-C (closed)
β3-T202
α2-R66
β3-E155
GABA
α
I
Distance Along Pore (Å)
9'
α2NB25
6HUK (closed 9' gate)
6HUO (open 9' gate)
Pore Radius (Å)
J
α
N62
α2NB25 FR3
N81
D122
Y59
α2NB25 FR2
E46
G47
Q121
R45
T202
β
Loop-C
K
WT
A201Y
T202Y
Specific binding (AU x 10³)
[α2NB25] (μM)
L
α2 β3-A201Y γ2
Co App
Post App
Current response (% GABA EC75)
[α2NB25] (μM)
M
A201Y
W116
T202
A201Y
CDR3
Loop-C
Non-clash rotamer
P114
N
T202Y
FW2
Non-clash rotamer
R45
T202Y
Loop-C
W116
A201
V110
F200
CDR3
O
ns
sIPSC amplitude (-pA)
Ctrl
α2NB25
P
ns
sIPSC frequency (Hz)
Ctrl
α2NB25
Q
ns
sIPSC rise time (ms)
Ctrl
α2NB25
R
ns
GABAA Itonic (-pA)
Ctrl
α2NB25
S
ns
GABAA RMS noise (pA)
Ctrl
α2NB25

Fig. S4. Functional and structural properties of α2$_{NB}$25. (**A**) α2$_{NB}$25 dose-response curves for inhibition of EC_{75} and EC_{15} GABA responses on recombinant α2β3γ2 during co-application versus for the subsequent GABA alone application. Mean ± S.E.M. (n = 3). (**B**) Data points comparing the pIC_{50} for co-application and post-application showing that post-application of GABA is more sensitive to inhibition by α2$_{NB}$25 for both EC_{75} and EC_{15} GABA applications. Mean ± S.E.M. (n = 3 cells). P values (two-sided) were calculated by paired Student's t-test; *, $P < 0.05$. (**C**) CDR3 electron density, protein sequence and model build for α2$_{NB}$25. Nitrogen atoms – blue, oxygen atoms – red. (**D**) $GABA_AR$ α2 ECD ribbon representation showing side chain atoms for residues that interface with α2$_{NB}$25 (cutoff < 3 Å). Residues in grey slate colour are different in the α1 subunit, providing the molecular basis for α2$_{NB}$25 α-subtype selectivity. (**E**) Specific binding curves for α2$_{NB}$25 to α2β1, α2β2, α2β3 receptors. Mean ± S.E.M. (n = 6 separate experiments). (**F**) View of ribbon representation of β3-subunit (blue) and C_α backbone representation of α2$_{NB}$25 (rainbow color from blue, N-terminus, to red, C-terminus) overlaid by a structural alignment for the equivalent regions of the α2β3+α2$_{NB}$06 model (grey). α2$_{NB}$06 exhibits a similar binding mode, with double headed curved arrows indicating a slight tilt meaning this NB is positioned slightly further away from loop-C allowing it to close inwards and bind GABA, versus α2$_{NB}$25 which interacts with loop-C to stabilise it in an outward conformation that does not bind GABA. (**G**) Cross-section at the 9′ Leu ring superposed by the antagonist bicuculline-bound α1β3γ2 model (PDB: 6HUK, grey) showing both models have leucines facing into the pore. (**H**) Ribbon representation of α2$_{NB}$25 CDR3 (yellow to orange) bound across the α2 (light pink)/β3 (blue) interface, superposed with the equivalent α2/β3 region from the GABA-bound α2β3γ2+α2$_{NB}$04 model (grey with GABA shown in magenta), showing that α2$_{NB}$25 CDR3 does not directly overlap with the GABA binding pose. Nitrogen atoms – blue, oxygen atoms – red. (**I**) Pore radius plot. Inhibited α1β3γ2 receptor (bound by antagonist bicuculline) is PDB 6HUK; GABA-bound α1β3γ2 receptor (also bound by alprazolam) is PDB 6HUO. (**J**) α2$_{NB}$25 FR2 and FR3 bound across the top of the α2 subunit. Nitrogen atoms – blue, oxygen atoms – red. A structurally aligned loop-C overlay from GABA-bound α1β3γ2 (PDB: 6HUO) is shown in grey for comparison. Curved arrow indicates α2$_{NB}$25 CDR3 forcing loop-C outwards – the interfacial contacts from α2$_{NB}$25 CDR3 are not shown for clarity (see Fig. 3E). (**K**) Specific binding curve for α2$_{NB}$25 to α2β3γ2 receptors, wild-type versus β3-A201Y or T202Y mutations in the presence of 500 μM GABA. Mean ± S.E.M. (n = 3 separate experiments). (**L**) α2$_{NB}$25 dose-response curve for inhibition of EC_{75} GABA responses on recombinant α2β3-A201Y-γ2 receptors. Mean ± S.E.M. (n = 4 cells). (**M-N**) Structural model close-ups for the $GABA_A$ receptor β-subunit loop-C interaction with α2$_{NB}$25 CDR3, presenting alternative rotamers for (**M**) an A201Y mutation or (**N**) a T202Y mutation, revealing that some rotamers can be accommodated without causing steric clashes, explaining why these two mutations do not reduce α2$_{NB}$25 affinity. (**O-S**) Histograms measured from DGGCs before and after incubation with 3 μM α2$_{NB}$25, for (**O**) sIPSC amplitudes, (**P**) sIPSC frequency, (**Q**) sIPSC rise time, (**R**) tonic current, (**S**) RMS tonic noise. Mean ± S.E.M. (n = 8 cells). p values (two-sided) were calculated by paired Student's t-test; ns, $P > 0.05$.

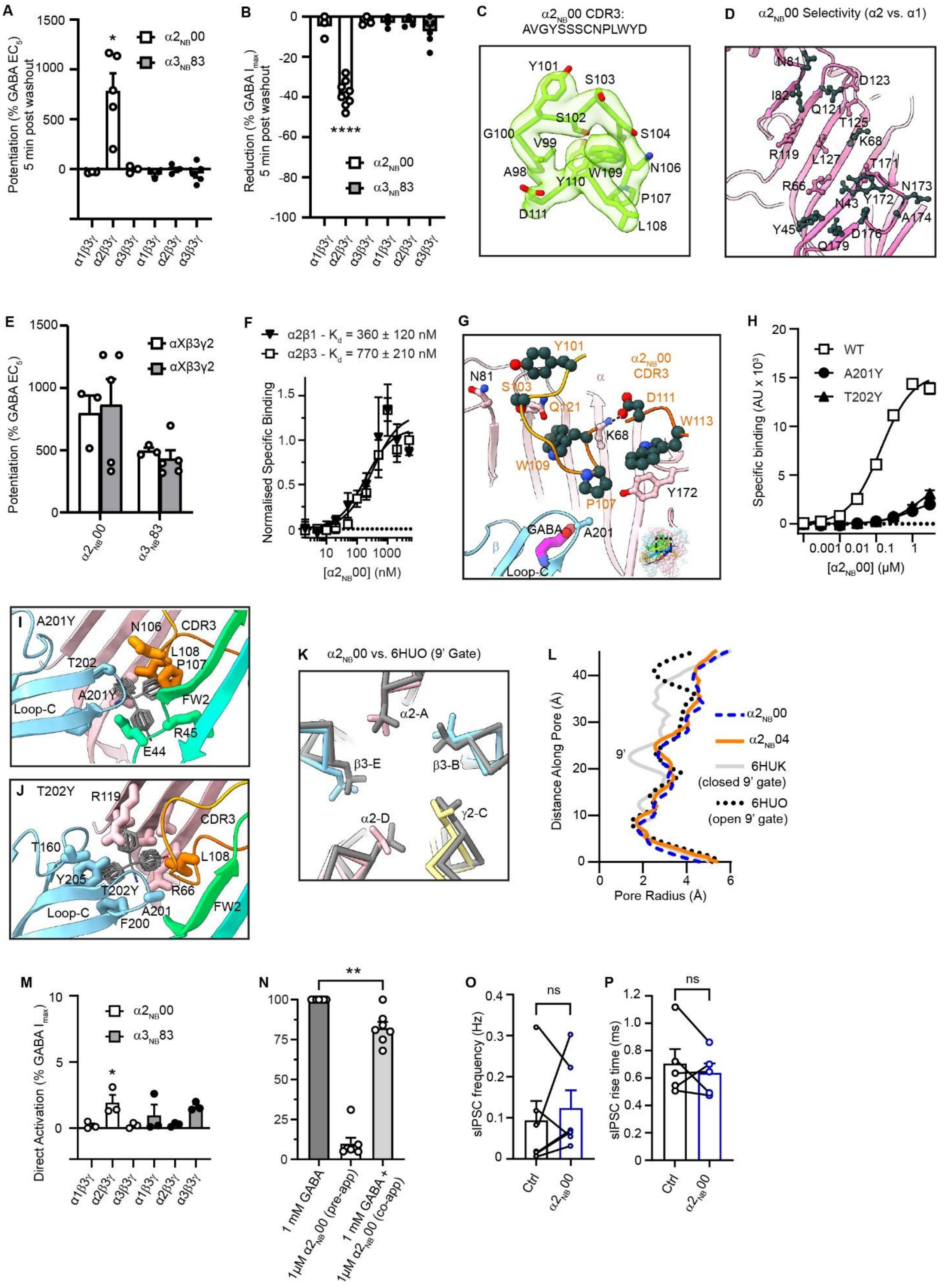

A
B
C
α2NB00 CDR3:
AVGYSSSCNPLWYD
D
α2NB00 Selectivity (α2 vs. α1)
E
F
α2β1 - Kd = 360 ± 120 nM
α2β3 - Kd = 770 ± 210 nM
G
H
I
J
K
α2NB00 vs. 6HUO (9' Gate)
L
M
N
O
P

Fig. S5. Functional and structural properties of NB PAMs. (**A**) Histogram showing potentiation of EC_5 GABA responses at a time point 5 minutes after application of 3 μM NB. Mean ± S.E.M. (*n* = 3-6 cells). *P* values from Tukey's multiple comparison tests; *, $P < 0.05$. (**B**) Histogram showing reduction of GABA I_{max} responses at a time point 5 minutes after application of 3 μM NB. Mean ± S.E.M. (*n* = 3-8 cells). ****, $P < 0.001$. (**C**) CDR3 electron density, protein sequence and model build for $\alpha2_{NB}00$. Nitrogen atoms – blue, oxygen atoms – red. (**D**) $GABA_AR$ α2 ECD ribbon representation showing side chain atoms for residues that interface with $\alpha2_{NB}00$ (cutoff < 3 Å). Residues in grey slate colour are different in the α1 subunit, providing the molecular basis for $\alpha2_{NB}00$ α-subtype selectivity. (**E**) Histogram showing potentiation of EC_5 GABA responses by 3 μM NB for β2 versus β3-containing $GABA_ARs$. $\alpha_X = \alpha2$ for testing of $\alpha2_{NB}00$, and α3 for testing $\alpha3_{NB}83$. Mean ± S.E.M. (*n* = 3-5). (**F**) Specific binding curves for $\alpha2_{NB}00$ to α2β1 and α2β3 receptors. Mean ± S.E.M. (*n* = 6 separate experiments). (**G**) $\alpha2_{NB}00$ CDR3 bound across the top of the α2 subunit. Electrostatic interaction shown as dashed line. Nitrogen atoms – blue, oxygen atoms – red, GABA - magenta. (**H**) Specific binding curve for $\alpha2_{NB}00$ to α2β3γ2 receptors, wild-type versus β3-A201Y or T202Y mutations in the presence of 500 μM GABA. Mean ± S.E.M. (*n* = 3 separate experiments). (**I-J**) Structural model close-ups for the $GABA_A$ receptor β-subunit loop-C interaction with $\alpha2_{NB}00$ FR2/CDR3, presenting alternative rotamers for (**I**) an A201Y mutation or (**J**) a T202Y mutation, revealing that all rotamers cause steric clashes, explaining why these two mutations ablate $\alpha2_{NB}00$ binding. (**K**) Cross-section at the 9′ Leu ring for the $\alpha2_{NB}25$ bound α2β3γ2 receptor superposed by the GABA-bound α1β3γ2 model (PDB: 6HUO, grey) showing both models have leucines facing sideways away from the centre of the pore. (**L**) Pore radius plot. Inhibited α1β3γ2 receptor (bound by antagonist bicuculline) is PDB 6HUK; GABA-bound α1β3γ2 receptor is PDB 6HUO. (**M**) Histogram showing direct activation by application of 3 μM NB, in the absence of GABA. Mean ± S.E.M. (*n* = 3 cells). *, $p < 0.05$. (**N**) Histogram showing GABA I_{max} before and after 5 minute pre-incubation with 1 μM $\alpha2_{NB}00$ and then co-application of a saturating dose of 1 mM GABA + 1 μM $\alpha2_{NB}00$. Mean ± S.E.M. (*n* = 7 cells). **, $p < 0.01$. (**O-P**) Histograms measured from DGGCs before and after incubation with 3 μM $\alpha2_{NB}00$ for (**O**) sIPSC frequency, or (**P**) sIPSC rise time. Mean ± S.E.M. (*n* = 5 cells). *P* values (two-sided) were calculated by paired Student's t-test (rise time) or Wilcoxon matched-pairs signed rank test (frequency); ns, $P > 0.05$.

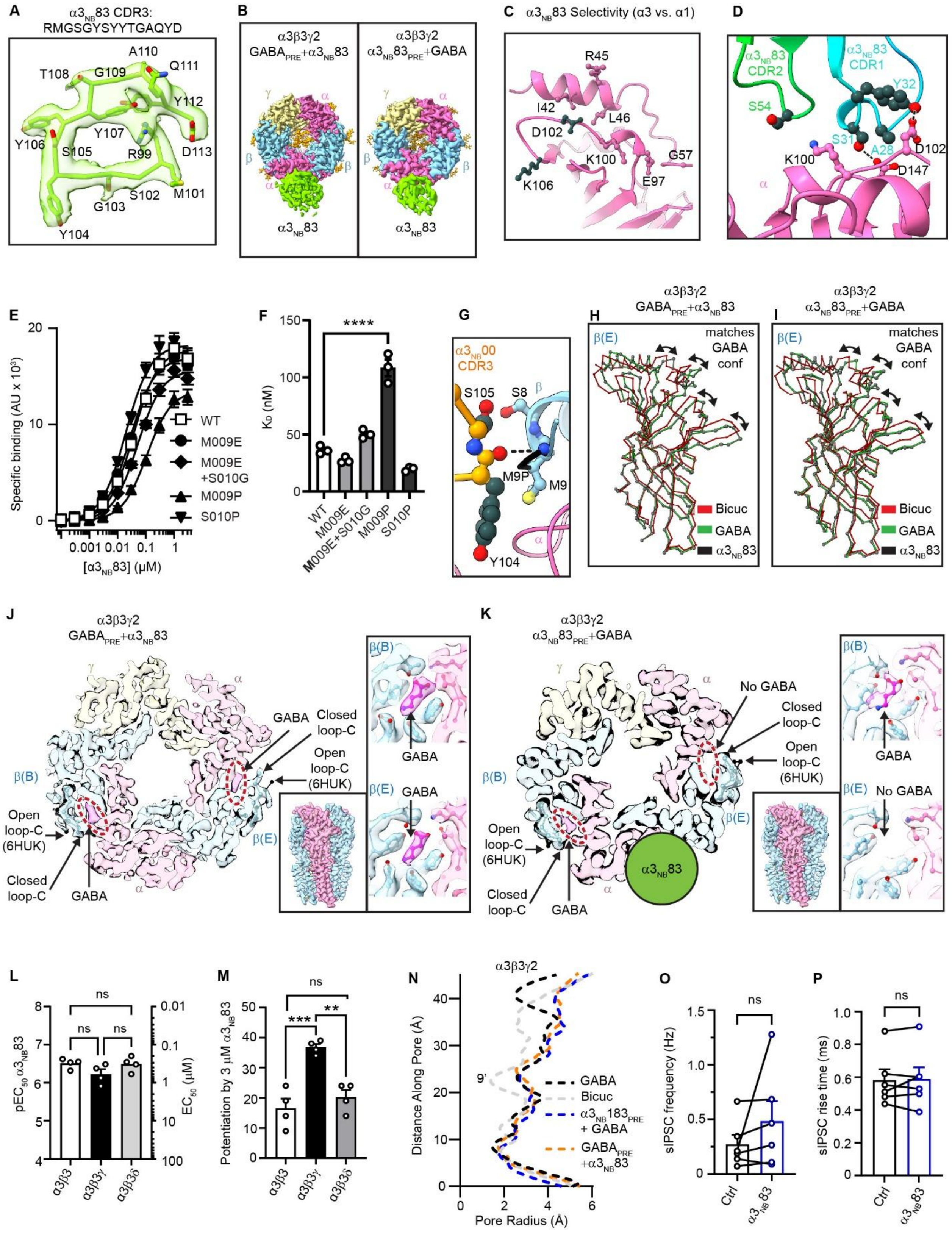
A
α3NB83 CDR3:
RMGSGYSYYTGAQYD
A110
T108
G109
Q111
Y112
Y106
Y107
S105
R99
D113
S102
M101
G103
Y104
B
α3β3γ2
GABAPRE+α3NB83
α3β3γ2
α3NB83PRE+GABA
γ
α
β
α3NB83
C
α3NB83 Selectivity (α3 vs. α1)
R45
I42
L46
D102
K100
G57
K106
E97
D
α3NB83
CDR2
α3NB83
CDR1
Y32
S54
S31
K100
A28
D102
D147
E
Specific binding (AU x 10³)
[α3NB83] (μM)
WT
M009E
M009E
+S010G
M009P
S010P
F
****
KD (nM)
WT
M009E
M009E+S010G
M009P
S010P
G
α3NB00
CDR3
S105
S8
M9P
M9
Y104
H
α3β3γ2
GABAPRE+α3NB83
β(E)
matches
GABA
conf
Bicuc
GABA
α3NB83
I
α3β3γ2
α3NB83PRE+GABA
β(E)
matches
GABA
conf
Bicuc
GABA
α3NB83
J
α3β3γ2
GABAPRE+α3NB83
GABA
Closed
loop-C
Open
loop-C
(6HUK)
β(B)
β(E)
Open
loop-C
(6HUK)
Closed
loop-C
GABA
K
α3β3γ2
α3NB83PRE+GABA
No GABA
Closed
loop-C
Open
loop-C
(6HUK)
β(B)
β(E)
Open
loop-C
(6HUK)
Closed
loop-C
GABA
No GABA
α3NB83
L
pEC50 α3NB83
EC50 (μM)
ns
α3β3
α3β3γ
α3β3δ
M
Potentiation by 3 μM α3NB83
ns
***
**
α3β3
α3β3γ
α3β3δ
N
α3β3γ2
Distance Along Pore (Å)
Pore Radius (Å)
9'
GABA
Bicuc
α3NB183PRE
+ GABA
GABAPRE
+α3NB83
O
sIPSC frequency (Hz)
ns
Ctrl
α3NB83
P
sIPSC rise time (ms)
ns
Ctrl
α3NB83

Fig. S6. Functional and structural properties of α3$_{NB}$83 PAM. (**A**) CDR3 electron density, protein sequence and model build for α3$_{NB}$83. Nitrogen atoms – blue, oxygen atoms – red. (**B**) Top-down view of α3β3γ2 cryo-EM maps (β3s – blue; α2s – hot pink; γ2 khaki) bound by α3$_{NB}$83 (green) showing clear density for only one bound NB copy, for two separate structures, one preincubated with GABA before adding NB, α2β3γ2+GABA$_{PRE}$+α3$_{NB}$83 (left panel), the other preincubated with NB before adding GABA, α2β3γ2+α3$_{NB}$83$_{PRE}$+GABA (right panel). (**C**) GABA$_A$R α2 ECD ribbon representation showing side chain atoms for residues that interface with α3$_{NB}$83 (cutoff < 3 Å). Residues in grey slate colour are different in the α1 subunit, providing the molecular basis for α3$_{NB}$83 α-subtype selectivity. (**D**) α3$_{NB}$83 CDR1 and CDR2 bound across the top of the α3 subunit. Nitrogen atoms – blue, oxygen atoms – red. The interfacial contacts from α2$_{NB}$25 CDR3 are not shown for clarity (see **Fig. 6F**). (**E**) Specific binding curve for α3$_{NB}$83 to α3β3γ2 receptors, wild-type versus β3-M009E, M009E+S010G, M009P or S010P mutations in the presence of 500 μM GABA. Mean ± S.E.M. (*n* = 3 separate experiments). (**F**) Histogram of the specific binding K$_d$s from (**E**). Mean ± S.E.M. (*n* = 3 separate experiments). Mean ± S.E.M. (*n* = 4-8). *P* values calculated by non-parametric one-way ANOVA with Tukey's multiple comparisons. ****, *P* < 0.001. (**G**) Structural model close-up for the GABA$_A$ receptor β-subunit α-helix interaction with α3$_{NB}$83 CDR3, showing the M9 side chain and an overlaid alternative proline mutation (black) to show how the proline mutation could alter the backbone Y104 carbonyl to M9 amide interaction, to explain the 3-fold reduction in binding affinity caused by this mutation. (**H-I**) Global pentamer alignment and subsequent C$_α$ model view of the β-subunit chain E that contacts α3$_{NB}$83 (not shown) for (**H**) α3β3γ2+GABA$_{PRE}$+α3$_{NB}$83 and (**I**) α3β3γ2+α3$_{NB}$83$_{PRE}$+GABA. The two α3$_{NB}$83 bound β-subunits are shown in black versus the bicuculline-bound α3β3γ2 + silent α2$_{NB}$77 fiducial structure (red, PDB:6HUK), and GABA-bound α3β3γ2 + silent α2$_{NB}$77 fiducial structure (green, PDB:6HUO), showing the upper ECD is rotated to match the GABA-bound conformation. (**J-K**) Cryo-EM map views of GABA pockets for (**J**) α3β3γ2+GABA$_{PRE}$+α3$_{NB}$83 and (**K**) α3β3γ2+α3$_{NB}$83$_{PRE}$+GABA. Slice view through the α3$_{NB}$83-bound α3β3γ2 receptor showing GABA density (magenta) present in both pockets for α3β3γ2+GABA$_{PRE}$+α3$_{NB}$83 but absent from the β(E) pocket for α3β3γ2+α3$_{NB}$83$_{PRE}$+GABA (pocket indicated by red dashed ovals), even though both loop-Cs are closed in both models, as shown by comparison to superposed open loop-Cs from bicuculline-bound α1β3γ2 (black, PDB: 6HUK). Insets show close-ups of the two neurotransmitter pockets, and also a whole pentamer side view to show contour level used for the pockets and top-down slice view. (**L**) Histogram showing the α3$_{NB}$83 pEC$_{50}$ values for potentiation of EC$_{10}$ GABA responses from α3β3, α3β3γ2 and α3β3δ receptors. (*n* = 4 cells). *P* values from Tukey's multiple comparison tests; ns, *P* > 0.05. (**M**) Histogram showing the α3$_{NB}$83 maximal potentiation of EC$_{10}$ GABA responses from α3β3, α3β3γ2 and α3β3δ receptors. (*n* = 4 cells). *P* values from Tukey's multiple comparisons test; ns, *P* > 0.05, **, *P* < 0.01, ***, *P* < 0.001. (**N**) Pore radius plot. Inhibited α1β3γ2 receptor (bound by antagonist bicuculline) is PDB 6HUK; GABA-bound α1β3γ2 receptor is PDB 6HUO. (**O-P**) Histograms measured from DGGCs before and after incubation with 3 μM α3$_{NB}$83, for (**O**) sIPSC frequency, or (**P**) sIPSC rise time. Mean ± S.E.M. (*n* = 5 cells). *P* values (two-sided) were calculated by paired Student's t-test; ns, *P* > 0.05.

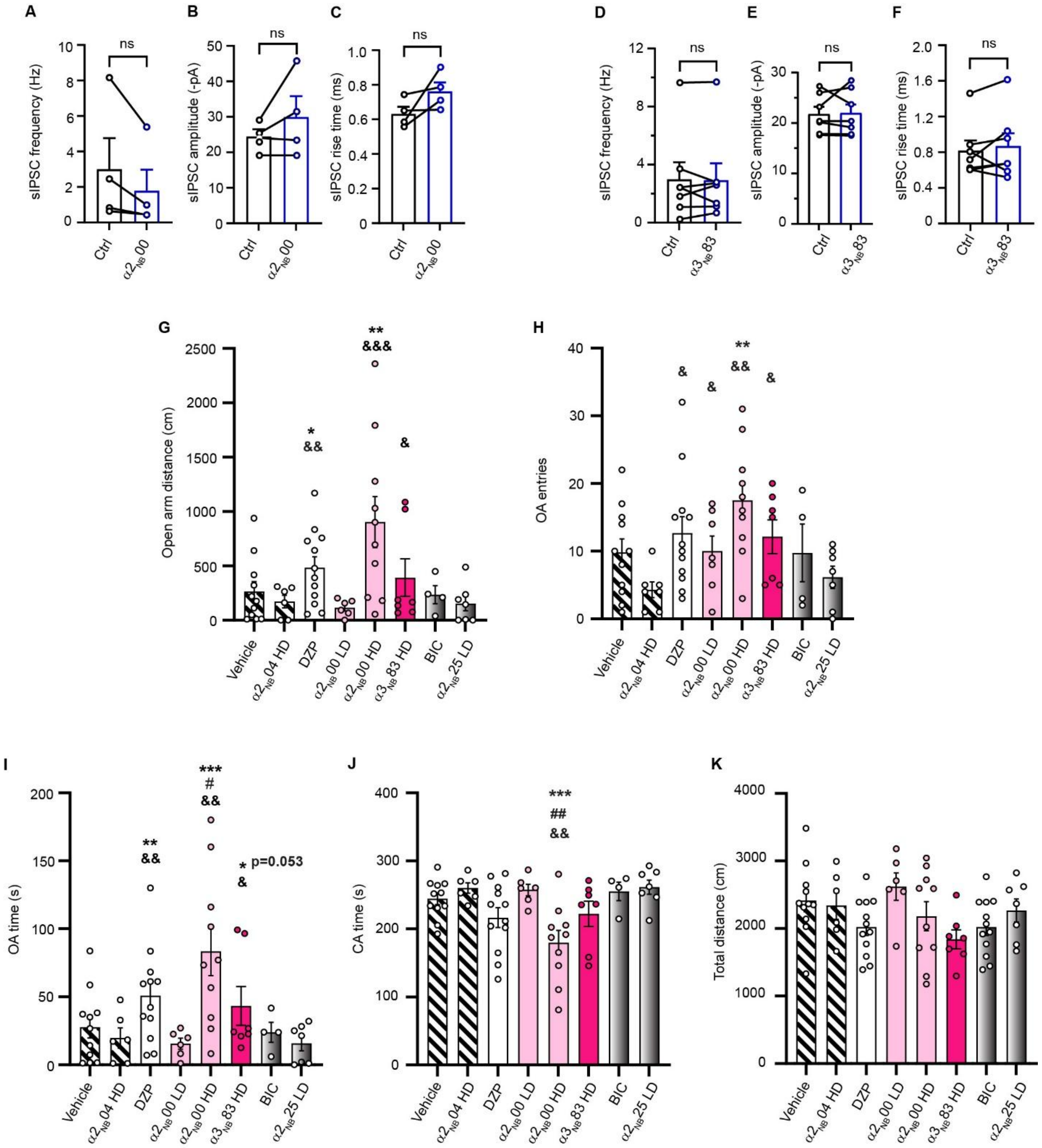
A
B
C
D
E
F
ns
sIPSC frequency (Hz)
sIPSC amplitude (-pA)
sIPSC rise time (ms)
Ctrl
α2NB00
α3NB83
G
H
Open arm distance (cm)
OA entries
Vehicle
α2NB04 HD
DZP
α2NB00 LD
α2NB00 HD
α3NB83 HD
BIC
α2NB25 LD
I
J
K
OA time (s)
CA time (s)
Total distance (cm)
p=0.053

Fig. S7. Electrophysiological and behavioural measurements for NB effects on the amygdala. (**A-F**) Histograms measured from basolateral amygdala principal neurons before and after incubation with 3 µM $\alpha2_{NB}00$ for (**A**) sIPSC frequency, (**B**) sIPSC amplitude, (**C**) sIPSC rise time, or with $\alpha3_{NB}83$ for (**D**) sIPSC frequency, (**E**) sIPSC amplitude, (**F**) sIPSC rise time. Mean ± S.E.M. (*n* = 4 cells). *P* values (two-sided) were calculated by paired Student's t-test or Wilcoxon matched-pairs signed rank test (frequency for both and $\alpha3_{NB}83$ rise time); ns, $P > 0.05$. (G-K) Histograms from behaviour testing in EPM for (**G**) distance travelled on the open arms (OA), (**H**) number OA entries, (**I**) time spent on OAs, (**J**) time spent in closed arms, (**K**) total distance travelled. HD – High dose (250 µM); LD – Low dose (40 µM); DZP – Diazepam (14 mM); BIC – Bicuculline (2.7 mM). Mean ± S.E.M (*n* = 4-12 per treatment group). *, $P < 0.05$; **, $P < 0.01$; ***, $P < 0.001$; planned comparisons vs pooled controls (vehicle and silent NB groups combined). #, $P < 0.05$; ##, $P < 0.01$; planned comparisons vs vehicle control only. &, $P < 0.05$; &&, $P < 0.01$; &&&, $P < 0.001$; planned comparisons versus silent $\alpha2_{NB}04$ binder control.

Table S1. α2 and α3 NBs in media binding scores (% versus direct strep staining of SBP-fused receptors) to each α-subtype in αXβ3 receptors. Scores above 5 % for off-target α-subtype binding are shown in red bold underline. *n* = 3.

α2NB #	CDR3 family	α1	α3	α4	α5	α6	α2
**46**		2	2	1	1	1	144
**17**		2	0	0	2	2	183
**05**	1	1	1	1	1	1	199
**04**		1	0	0	2	0	240
**47**		1	2	1	1	1	110
**96**		0	0	0	0	0	109
**97**	2	1	0	1	0	1	85
**98**		0	0	1	1	1	48
**00**		2	1	0	1	1	66
**99**	3	0	0	0	1	0	52
**01**		0	0	0	0	0	58
**07**		1	2	1	0	1	54
**08**	4	1	1	1	1	1	14
**20**		1	1	1	0	1	33
**21**	5	1	2	2	1	0	28
**22**		0	0	0	0	0	13
**30**	6	0	0	1	0	1	15
**25**	7	0	0	0	0	2	146
**06**	8	1	0	1	0	1	131
**13**	11	0	1	1	0	1	18
**14**	12	0	0	0	0	2	99
**15**	13	0	0	1	0	1	47
**16**	14	0	0	0	0	0	193
**18**	15	0	0	1	0	0	210
**19**	16	0	0	1	0	0	208
**23**	17	0	0	1	1	0	27
**24**	18	0	0	0	0	1	10
**27**	19	1	2	0	1	1	51
**29**	21	**7**	0	0	1	2	85
**31**	22	0	0	1	0	0	13
**32**	23	0	0	1	0	0	29
**33**	24	0	0	0	0	0	24
**36**	27	0	0	0	1	0	92
**37**	28	0	1	0	0	1	113
**38**	29	0	1	2	1	1	14
**41**	31	**15**	1	2	0	0	8
**42**	32	1	1	1	1	1	35
**44**	34	1	2	1	0	0	36
**45**	35	1	2	2	1	0	71
**48**	36	1	1	2	1	1	116
**49**	37	1	1	1	0	0	48
**52**	38	1	2	1	2	1	109
**α3NB #**	**CDR3 family**	**α1**	**α2**	**α4**	**α5**	**α6**	**α3**
**77**		3	2	1	3	1	132
**78**		2	2	1	4	1	179
**61**		4	0	0	2	1	35
**62**		2	0	0	0	1	45
**59**		4	0	1	2	1	24
**54**	1	2	0	2	4	5	87
**53**		2	0	2	3	1	31
**57**		3	0	0	2	1	16
**56**		2	0	1	2	**25**	133
**58**		3	0	0	2	0	127
**87**	3	1	1	3	3	1	18
**73**	4	4	3	1	**90**	0	16
**80**	8	2	2	0	4	1	15
**81**	9	0	0	0	0	0	16
**83**	11	1	1	0	1	0	18
**04**	26	1	1	1	1	1	17

Table S2. NB on-cell binding affinities. "–" denotes experiment not done. *n* = 3.

NB Number	Dissociation Constant, K_D (nM) +GABA	Dissociation Constant, K_D (nM) -GABA
$\alpha2_{NB}46$	210 ± 93	–
$\alpha2_{NB}05$	205 ± 28	–
$\alpha2_{NB}04$	270 ± 36	–
$\alpha2_{NB}47$	370 ± 18	–
$\alpha2_{NB}00$	892 ± 275	1814 ± 751
$\alpha2_{NB}25$	2,309 ± 778	2391 ± 697
$\alpha2_{NB}06$	2,763 ± 520	–
$\alpha2_{NB}16$	409 ± 188	–
$\alpha2_{NB}29$	693 ± 117	–
$\alpha3_{NB}77$	37 ± 2	–
$\alpha3_{NB}56$	58 ± 20	–
$\alpha3_{NB}83$	139 ± 25	747 ± 191

Table S3. Cryo-EM data collection, refinement, and validation statistics for $GABA_AR$ datasets.

Data Collection	α2β3 + $α2_{NB}06$	α2β3 + $α2_{NB}16$	α2β3 + $α2_{NB}29$	α2β3 + $α2_{NB}47$	α3β3 + $α3_{NB}77$
PDB Code	PDB-9QK1	PDB-9QJS	PDB-9QJR	PDB-9QJV	PDB-9QJQ
EMDB Code	EMDB-53216	EMDB-53209	EMDB-53208	EMDB-53212	EMDB-53207
Facility	Biochemistry Cryo-EM Facility, University of Cambridge	Biochemistry Cryo-EM Facility, University of Cambridge	Biochemistry Cryo-EM Facility, University of Cambridge	Biochemistry Cryo-EM Facility, University of Cambridge	Biochemistry Cryo-EM Facility, University of Cambridge
Microscope	Titan Krios	Titan Krios	Titan Krios	Titan Krios	Titan Krios
Magnification	130k	130k	130k	130k	130k
Voltage (kV)	300	300	300	300	300
Exposure Time (s)	10	1.61	1.45	12	1.20
Number of Frames	380	120	108	480	89
Electron Exposure (e^-/Å)	51.50	47.84	49.27	58.38	55.55
Detector	Gatan K2	Gatan K3	Gatan K3	Gatan K2	Gatan K3
Pixel Size (Å/pixel)	1.07	0.65	0.65	1.05	0.63
Micrographs	1,173	2,871	2,253	587	2,047
**Reconstruction**					
Initial Number of Particles	312,451	592,056	351,112	229,313	394,331
Final Number of Particles	142,272	344,106	192,924	30,394	102,406
Symmetry Imposed	C1	C1	C1	C1	C1
Box Size (pixels)	370	520	520	320	440
Map Resolution (Å)	3.05	2.52	2.92	3.45	2.63
FSC Threshold	0.143	0.143	0.143	0.143	0.143
Map Sharpening B Factor (A^2)	81.0	76.9	88.4	59.5	54.1
**Refinement**					
Number of Non-H Atoms	17,635	17,655	17,827	17,672	17,819
Protein Residues	2,140	2,128	2,156	2,135	2,148
N-Glycans	26	34	33	30	37
Ligands	4	4	4	4	4
Water	0	0	0	0	1
MolProbity Score	1.07	1.20	1.18	1.38	1.54
Clashscore	2.80	4.17	3.88	3.65	7.49
Poor Rotamers (%)	0.11	0.16	0.16	0.05	0.11
Bond Length (Å) RMSD	0.002	0.003	0.002	0.004	0.004
Bond Angle (°C) RMSD	0.408	0.431	0.399	0.426	0.529
Ramachandran Favoured (%)	98.20	98.43	98.31	96.58	97.31
Ramachandran Outliers (%)	0.00	0.00	0.00	0.00	0.00

Table S4. Cryo-EM data collection, refinement, and validation statistics for $GABA_AR$ datasets.

**Data Collection**	α2β3γ2 + $α2_{NB}04$	α2β3γ2 + $α2_{NB}25$	α2β3γ2 + $α2_{NB}00$	α3β3γ2 + $α3_{NB}83_{PRE}$ + GABA	α3β3γ2 + $GABA_{PRE}$ + $α3_{NB}83$	α3β3γ2 ++ GABA + $α3_{NB}77$	α3β3γ2 ++ bicuculline + $α3_{NB}77$
PDB Code	PDB-9QJY	PDB-9QK0	PDB-9QJX	PDB-9QJP	PDB-9TNS	PDB – 9TPQ	PDB-9TNB
EMDB Code	EMDB-53214	EMDB-53215	EMDB-53213	EMDB-53206	EMDB-56094	EMDB-56109	EMDB-56081
Facility	Electron Bio-Imaging Centre (EBIC) UK	Electron Bio-Imaging Centre (EBIC) UK	Electron Bio-Imaging Centre (EBIC) UK	Electron Bio-Imaging Centre (EBIC) UK	UoC Biochem Facility (UK)	Electron Bio-Imaging Centre (EBIC) UK	Electron Bio-Imaging Centre (EBIC) UK
Microscope	Titan Krios	Titan Krios	Titan Krios	Titan Krios	Titan Krios	Titan Krios	Titan Krios
Magnification	130k	130k	130k	130k	165k	130k	130k
Voltage (kV)	300	300	300	300	300	300	300
Exposure Time (s)	1.32	1.22	1.32	1.30	4.39	1.55	1.55
Number of Frames	100	92	100	100	150	150	150
Electron Exposure (e^-/Å)	50.00	49.85	50.00	50.00	50.47	51.25	51.25
Detector	Gatan K3	Gatan K3	Gatan K3	Gatan K3	Falcon 4i	Gatan K3	Gatan K3
Pixel Size (Å/pixel)	0.65	0.65	0.65	0.65	0.73	0.65	0.65
Micrographs	3,043	17,489	8,354	6,481	1838	3739	4031
**Reconstruction**							
Initial Number of Particles	386,836	496,738	698,270	626,312	314,781	578,387	630,021
Final Number of Particles	170,826	70,609	297,968	94,612	81,900	89,390	152,562
Symmetry Imposed	C1	C1	C1	C1	C1	C1	C1
Box Size (pixels)	384	384	384	384	480	480	480
Map Resolution (Å)	2.78	3.01	2.70	3.22	2.75	2.94	2.87
FSC Threshold	0.143	0.143	0.143	0.143	0.143	0.143	0.143
Map Sharpening B Factor (A^2)	83.8	80.8	101.5	96.1	54.0	83.8	89.0
**Refinement**							
Number of Non-H Atoms	15,833	15,845	15,735	14,943	14761	15761	14721
Protein Residues	1,912	1,913	1,902	1791	1791	1894	1894
N-Glycans	31	31	31	33	33	33	33
Ligands	2	0	2	2	2	2	2
Water	0	0	0	0	0	0	0
MolProbity Score	1.46	1.38	1.06	1.48	1.14	1.34	1.31
Clashscore	4.38	3.72	2.08	4.54	2.02	2.8	2.71
Poor Rotamers (%)	0.12	0.35	0.18	0.13	0	0.12	0.18
Bond Length (Å) RMSD	0.004	0.003	0.002	0.002	0.003	0.003	0.003
Bond Angle (°C) RMSD	0.455	0.466	0.411	0.462	0.508	0.536	0.478
Ramachandran Favoured (%)	96.41	96.57	97.61	96.22	97.01	96.05	96.21
Ramachandran Outliers (%)	0.00	0.00	0.00	0.00	0.00	0.00	0.05

Table S5. α-subtype residue differences versus α2 or α3, within 3 Å of α2 or α3 nanobody

$\alpha2_{NB}25$					
**α2**	**α1**	**α3**	**α4**	**α5**	**α6**
F041	K042	K066	K040	R045	K040
K068	S069	T093	T067	S072	T067
K070		H095	I069		T069
F076			Y075		
K077		D102	D076		G076
G078					
N081	T082	K106	E080	Q085	E080
I082	V083			R086	
R119					
Q121	T122	V146	M120	E125	M120
D122	E123		R121		Q121
D123		N148	N122		N122
T125					
L127					
W170					
T171					K170
Y172	R173	L197	K171	N176	K171
D176	R177	K201	K175	K180	Y175
Q179	V180	E204	E178	V183	E178
**$\alpha2_{NB}00$**					
**α2**	**α1**	**α3**	**α4**	**α5**	**α6**
N043	D044	D068	D042	D047	D042
Y045	F046				
R066					
K068	S069	T093	T067	S072	T067
N081	T082	K106	E080	Q085	E080
I082	V083			R086	
R119					
Q121	T122	V146	M120	E125	M120
D123		N148	N122		N122
T125					
L127					
T171					K170
Y172	R173	L197	K171	N176	K171
N173	E172	G198	G172	G177	G172
A174	P175	K199	P173	S178	P173
D176	R177	K201	K175	K180	Y175
Q179	V180	E204	E178	V183	E178
**$\alpha3_{NB}83$**					
**α3**	**α1**	**α2**	**α4**	**α5**	**α6**
I042					
R045			S019	G024	N019
L046					
G057					
E097			K071		
K100				R079	
D102	K078	K077		K081	G076
K106	T082	N081	E080	Q085	E080
